# Adamts1 Exacerbates Post‐Myocardial Infarction Scar Formation via Mechanosensing of Integrin α8

**DOI:** 10.1002/advs.202504138

**Published:** 2025-09-27

**Authors:** Chun‐Yan Kong, Zhen Guo, Yu‐Lan Ma, Ming‐Yu Wang, Hai‐Yang Ni, Pan Wang, Wen‐Jun Qiu, En‐Gui Wang, Zhou Li, Zheng Yang, Bo Shen, Qi‐Zhu Tang

**Affiliations:** ^1^ Department of Cardiology Renmin Hospital of Wuhan University Wuhan 430060 P. R. China; ^2^ Hubei Key Laboratory of Metabolic and Chronic Diseases Wuhan 430060 P. R. China; ^3^ TaiKang Center for Life and Medical Sciences Wuhan University Wuhan 430071 P. R. China; ^4^ Institute of Myocardial Injury and Repair Zhongnan Hospital of Wuhan University Wuhan 430071 P. R. China; ^5^ Department of Cardiology Zhongnan Hospital of Wuhan University Wuhan 430071 P. R. China; ^6^ Beijing Key Laboratory of Micro‐Nano Energy and Sensor Beijing Institute of Nanoenergy and Nanosystems Chinese Academy of Sciences Beijing 101400 China

**Keywords:** A Disintegrin and Metalloproteinase with Thrombospondin Motifs 1, ITGα8, mechanotransduction, myocardial infarction, scar formation

## Abstract

‐Myocardial infarction (MI) remains a leading cause of morbidity and mortality worldwide, with post‐infarction cardiac remodeling, particularly excessive scar formation, representing a critical determinant of patient outcomes. However, the mechanistic pathways governing pathological scar formation remain incompletely understood. Here, we demonstrate that ADAMTS1 (A Disintegrin and Metalloproteinase with Thrombospondin Motifs 1), significantly upregulated in endothelial cells (ECs) following MI, plays a pivotal role in regulating cardiac fibroblast activation through a novel mechanotransduction pathway involving integrin α8 (ITGα8). Using EC‐specific ADAMTS1 overexpression and knockout mice combined with cardiac fibroblast‐specific ITGα8 deletion models, we found that ADAMTS1 overexpression exacerbates cardiac dysfunction and increases scar size, while ADAMTS1 deficiency provides cardioprotection. Mechanistically, ADAMTS1 modulates extracellular matrix stiffness through proteoglycan (PG) cleavage rather than direct protein interactions, which activates ITGα8 mechanosensing specifically in cardiac fibroblasts. Among integrin family members tested, ITGα8 shows selective responsiveness to ADAMTS1‐mediated mechanical cues, as confirmed by tunable‐stiffness hydrogel experiments and validated through comprehensive proteomic and functional analyses. ITGα8 deficiency rescues ADAMTS1‐induced cardiac dysfunction and reduces pathological scar formation. These findings reveal a previously unrecognized ADAMTS1‐ITGα8 mechanotransduction pathway, representing a promising therapeutic target for optimizing post‐infarction cardiac remodeling.

## Graphical abstract

Proposed mechanistic model of ADAMTS1‐ITGα8 mechanotransduction in post‐MI scar formation. Schematic illustration showing the mechanistic pathway by which endothelial cell (EC) derived Adamts1 regulates cardiac fibroblast activation through ITGα8‐mediated mechanotransduction following myocardial infarction (MI). Upon MI‐induced hypoxia, ADAMTS1 is upregulated and secreted by ECs. ADAMTS1 cleaves extracellular matrix proteoglycans (PGs), reducing matrix stiffness and generating mechanical signals that activate ITGα8 mechanosensing in cardiac fibroblasts. This leads to myofibroblast activation, increased collagen synthesis, and excessive scar formation, creating a pathological feedback loop. Adamts1, a disintegrin and metalloproteinase with thrombospondin motif 1; ITGα8, integrin subunit α8; MI, MI.

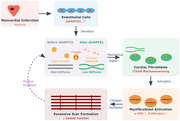

## 1 Introduction

Despite advances in risk management and diagnostic techniques, myocardial infarction (MI) remains a leading cause of morbidity and mortality globally.^[^ ^1^, ^2^ ^]^ The post‐infarction remodeling process involves a complex cascade of events regulated by various growth factors and cytokines, including transforming growth factor‐β (TGF‐β), connective tissue growth factor (CTGF), and vascular endothelial growth factor (VEGF), which modulate fibroblast activation, extracellular matrix (ECM) production, and angiogenesis.^[^ ^3^ ^]^ While cardiac fibrosis is initially adaptive, sustained profibrotic responses can lead to maladaptive scarring and chronic heart failure.^[^ ^4^, ^5^ ^]^ This highlights the critical need for understanding these mechanisms and developing targeted therapeutic strategies to improve long‐term prognosis in MI patients.

A Disintegrin and Metalloproteinase with Thrombospondin Motifs 1 (ADAMTS1) is a multifunctional extracellular protease that is highly conserved across mammalian species (>89% amino acid identity between human and mouse).^[^ ^6^ ^]^ It has emerged as a critical player in cardiac repair and remodeling following MI. As a key enzyme responsible for cleaving proteoglycans (PGs) involved in ECM reconstruction, ADAMTS1 shows marked upregulation in the post‐MI myocardium, particularly in endothelial cells (ECs).^[^ ^7^, ^8^, ^9^ ^]^ Importantly, ADAMTS1 exhibits well‐documented concentration‐dependent effects on angiogenesis and ECM remodeling, particularly through versican cleavage.^[^ ^10^, ^11^ ^]^Clinical studies demonstrate that ADAMTS1 mutations are associated with increased risk of fatal coronary disease and genetic heterogeneity in drug efficacy.^[^ ^12^ ^]^ Nevertheless, its precise role in regulating PGs during post‐MI scar formation remains to be elucidated.

Recent advances in mechanobiology have revealed that mechanical cues play equally important roles in regulating cellular behavior and tissue remodeling in the post‐infarct myocardium, where tissue stiffness changes directly influence fibroblast activation and ECM production.^[^ ^13^, ^14^ ^]^ Tissue mechanical properties, particularly rigidity, serve as critical regulatory signals that cells sense and respond to through specialized mechanosensors. Integrins (ITGs) serve as the primary mechanosensors, responsible for mediating crucial interactions between cardiac fibroblasts (CFs) and the ECM.^[^ ^15^, ^16^ ^]^ Within the integrin family, ITGα8 has emerged as a particularly mechanosensitive receptor that responds to changes in substrate rigidity, although its specific role in cardiac mechanotransduction remains poorly understood.

Concurrently, ADAMTS1 has been established as a key player in ECM remodeling through its proteolytic activity on PGs,^[^ ^17^, ^18^ ^]^ whereby it likely alters the mechanical properties and composition of the developing scar tissue. The intersection between ADAMTS1‐mediated ECM remodeling and integrin mechanosensing represents a potentially critical regulatory axis, yet the precise mechanisms by which this ECM remodeling influences scar formation and cardiac function post‐MI remain largely unexplored.

Despite the advances in cardiac care, the extent of scar formation remains a critical determinant of post‐MI prognosis. Previous studies have largely focused on direct cell‐cell signaling and paracrine factors in cardiac remodeling, while the role of ECM mechanical properties as signaling mediators has been underappreciated. This study investigates the role of ADAMTS1 in promoting post‐MI scar formation through a novel mechanotransduction mechanism. We reveal that ADAMTS1 alters scar tissue mechanical properties through PG cleavage, subsequently activating mechanosensitive ITGα8 signaling in CFs and leading to excessive scar formation. These findings identify a previously unrecognized ADAMTS1‐ITGα8 mechanotransduction pathway in post‐MI remodeling and highlight potential therapeutic targets for improving outcomes in patients with ischemic heart disease.

## 2 Results

### 2.1 Post‐MI Scar Formation was Characterized by Abundant ECM‐Collagen and Proteoglycan Alterations

**Figure 1:**
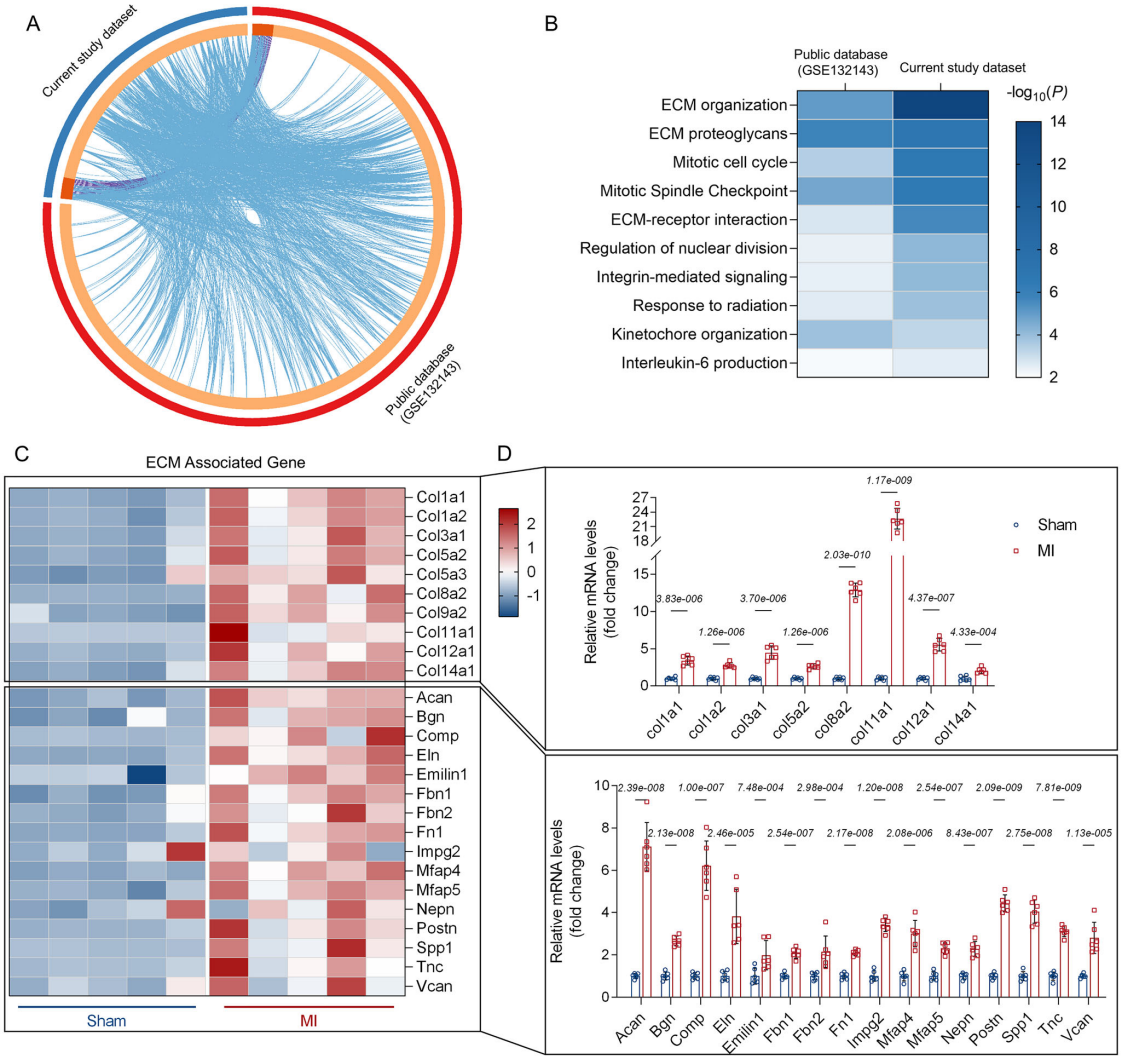
Collagen and proteoglycans (PGs) are abundant in extracellular matrix (ECM) of post‐MI infarct scar. A) Chord diagram showing pathway connections from integrated transcriptome analysis of human ischemic heart failure samples (GSE132143, *n* = 3) and murine MI models (*n* = 5). B) Heatmap depicting the top 10 enriched pathways from the integrated analysis. C) Heatmap depicting the expression of collagen and PG‐related genes in the ECM from murine transcriptome data (*n* = 5). D) Relative mRNA expression of collagen and PG‐related genes as determined using qRT‐PCR (*n* = 6 per group). Data are presented as mean ± SD. Statistical analysis was performed using multiple unpaired *t* tests with two‐stage step‐up method of Benjamini, Krieger, and Yekutieli for correction. *t* values for each gene are as follows: Col1a1 (9.223), Col1a2 (10.81), Col3a1 (9.419), Col5a2 (11.03), Col8a2 (31.41), Col11a1 (24.52), Col12a1 (12.77), Col14a1 (5.155), Acan (12.95), Bgn (13.68), Comp (10.78), Eln (5.314), Emilin1 (3.070), Fbn1 (9.512), Fbn2 (3.654), Fn1 (13.34), Impg2 (14.97), Mfap4 (7.344), Mfap5 (9.594), Nepn (8.230), Postn (20.08), Spp1 (12.56), Tnc (16.32), and Vcan (5.925). Abbreviations: Acan, Aggrecan; Bgn, Biglycan; Col, Collagen; Comp, Cartilage Oligomeric Matrix Protein; Eln, Elastin; Emilin1, Elastin Microfibril Interfacer 1; Fbn, Fibrillin; Fn1, Fibronectin 1; Impg2, Interphotoreceptor Matrix Proteoglycan 2; Mfap, Microfibril Associated Protein; MI, Myocardial Infarction; Nepn, Nephrocan; Postn, Periostin; Spp1, Secreted Phosphoprotein 1; Tnc, Tenascin C; Vcan, Versican.

Scar formation is a prerequisite for preserving cardiac structure and function following MI. The extent of scarring serves as an independent predictor of infarct prognosis, wherein excessive scar formation is associated with poor outcomes.^[^ ^19^ ^]^ To elucidate the molecular mechanisms regulating scar formation, we conducted a comparative analysis of transcriptional profiles from mouse scar tissue and heart tissue of ischemic heart failure patients. Integrated transcriptome analysis comparing the dataset from the current study with a public database (GSE132143) revealed significant alterations in ECM structural organization and PGs accumulation within the infarct area (IA) (**Figure** **1**A,B). Moreover, quantitative real‐time PCR (qRT‐PCR) analysis of genes related to ECM organization and ECM PGs pathways confirmed substantial collagen deposition (such as col1a1 and col3a1) at the scar site, accompanied by elevated expression of PG‐associated genes, such as ACAN and BGN (Figure 1C,D, Table S2, Supporting Information). Furthermore, expression patterns associated with adamalysins, matrix PGs, and collagen formation in the cardiac tissue transcriptome 14 days post‐MI were also observed (Figure S1A, Supporting Information). These were consistent with their hypothesized functions in ECM organization and PG accumulation. Adamalysins—comprising ADAM metalloproteinases—play a crucial role in ECM remodeling through PG cleavage.^[^ ^20^, ^21^ ^]^ Adamts1, 4, and 5 are the primary cardiac isoforms of adamalysins and possess high sequence homology (Figure S1B,C, Supporting Information). Based on these findings, this study sought to investigate the impact of Adamts1 on post‐MI scar formation.

### 2.2 Adamts1 Expression Increased Post‐MI, Predominantly in Endothelial Cells

**Figure 2:**
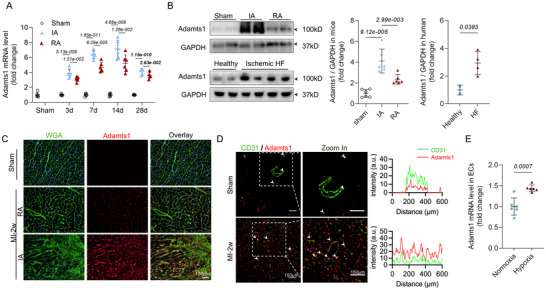
Adamts1 is upregulated in ECs post‐MI. A) Transcript level of *ADAMTS1* at different time points after myocardial infarction (MI) (*n* = 6 per group). 3 days: F (2,15) = 88.95, *p* = 4.79e‐009, 7 days: F (2, 15) = 191.1, *p* = 2.13e‐011, 14 days: F (2, 15) = 62.55, *p* = 5.28e‐008. 28 days: F (2, 15) = 160.8, *p* = 7.36e‐011. B) Western blot analysis of Adamts1 protein expression in mice with sham or MI surgery [*n* = 6 per group, F (2,15) = 26.12, *p* = 1.30e‐005], as well as healthy donors (*n* = 2) and patients with ischemic HF (*n* = 4, *t* = 3.038). C) Immunofluorescence (IF) staining showing the expression and localization of Adamts1 in cardiac tissue of mice 2 weeks post‐MI. D) In situ immunofluorescence (ISI) staining demonstrating co‐localization of Adamts1 with endothelial cells (ECs) (CD31, EC biomarker), with quantitative analysis of Adamts1/CD31 co‐localization coefficient in Sham and MI‐2W groups (*n* = 6 per group). E) Transcript level of *ADAMTS1* in ECs under normoxic and hypoxic conditions (*n* = 6, *t* = 4.796). Statistical analysis was performed using one‐way ANOVA followed by Bonferroni post hoc test (A,B) and unpaired *t* tests (B,E). Data are presented as the mean ± SD. Adamts1, a disintegrin and metalloproteinase with thrombospondin motif 1; IA, infarct area; RA, remote infarct area; HF, heart failure; WGA, wheat germ agglutinin; MI, myocardial infarction.

To elucidate the role of Adamts1 in post‐MI cardiac remodeling, we first examined cardiac tissue morphological changes (Figure S2A, Supporting Information) and then analyzed *ADAMTS1* transcriptional profiles at various time points following MI induction. *ADAMTS1* mRNA levels were significantly upregulated in both the IA and remote area (RA) compared to sham controls, showing peak expression at 14 days post‐MI followed by gradual decline but sustained elevation through 28 days, with consistently higher levels in the IA (**Figure** **2**A). Consistently, Adamts1 protein expression was significantly higher in heart tissue from mice 14 days post‐MI and in patients with ischemic heart failure (Figure 2B,C). The concordance between elevated *ADAMTS1* mRNA and protein expression, along with enhanced fibroblast activation markers (Figure S2B,C, Supporting Information), suggests Adamts1 involvement in scar formation and myofibroblast activation.

To determine the cellular source of Adamts1, we utilized a single‐cell omics database (CardiovascuLAR Atlas; https://clara.baker.edu.au/) and found that *ADAMTS1* was predominantly expressed in ECs and CFs, with significant and consistent upregulation in ECs post‐MI (Figure S2D, Supporting Information). Hypoxic stimulation, which mimics the post‐MI microenvironment, significantly increased *ADAMTS1* transcript levels in both human umbilical vein endothelial cells (HUVECs) and CFs (Figure S2E, Supporting Information). Immunofluorescence (IF) staining confirmed the predominant localization of Adamts1 in ECs, with quantitative co‐localization analysis demonstrating strong overlap with the endothelial marker CD31 (Figure 2D). Under hypoxic conditions, Adamts1 was prominently localized to extracellular and perimembrane regions in both HUVECs and CFs (Figure S2F, Supporting Information), consistent with its function as a secreted metalloproteinase. In vivo, Adamts1 expression was markedly upregulated in ECs within both the IA and under hypoxic conditions (Figure 2E). These findings establish that Adamts1 is predominantly expressed by ECs and is significantly upregulated in the post‐MI heart, positioning it as a key player in cardiac remodeling.

### 2.3 Validation of *ADAMTS1* Overexpression and Endothelial Cell Functional Characterization

To confirm the efficiency and specificity of endothelial‐targeted *ADAMTS1* overexpression, ADAMTS1^Tie^ and VEC^Tie^ control mice were administered tamoxifen 7 days prior to experimental procedures to ensure efficient gene expression modulation. We then performed comprehensive validation using multiple approaches. IF analysis demonstrated specific Adamts1 expression in ECs, with strong co‐localization with the endothelial marker CD31 in ADAMTS1^Tie^ mice compared to VEC^Tie^ controls (Figure S3A, Supporting Information). Western blot analysis confirmed significant Adamts1 protein upregulation in ADAMTS1^Tie^ mice (Figure S3B, Supporting Information). Cell‐type specific validation using isolated cardiac cell populations revealed that *ADAMTS1* mRNA levels were significantly elevated in ECs from ADAMTS1^Tie^ mice, while CFs and cardiomyocytes showed minimal changes, confirming endothelial specificity of our genetic modification (Figure S3C, Supporting Information). Western blot analysis of isolated cell populations further confirmed endothelial‐specific Adamts1 protein upregulation, with strong expression in ECs and minimal expression in cardiomyocytes and CFs from ADAMTS1^Tie^ mice (Figure S3D, Supporting Information). These findings confirm the endothelial specificity of our genetic modification at both transcriptional and protein levels.

To further validate the functional consequences of genetic *ADAMTS1* modification in ECs, comprehensive functional assays were performed using cardiac ECs isolated from the VEC^Tie^ and Adamts1^Tie^ mice. Tube formation assays revealed that *ADAMTS1*‐overexpressing ECs exhibited significantly reduced angiogenic capacity, along with decreased total tube length and reduced junction formations compared to the controls (Figure S4A,B, Supporting Information). In contrast, cell viability assays demonstrated no significant differences between the ADAMTS1^Tie^ and VEC^Tie^ ECs across all time points tested, thereby confirming that the observed functional changes were not due to cytotoxic effects (Figure S4C, Supporting Information). Furthermore, wound healing assays demonstrated significantly impaired EC migration in the Adamts1^Tie^ cells, whereas cell viability remained unchanged across all time points tested (Figure S4D,E, Supporting Information). These findings confirm that genetic *ADAMTS1* modification produces functionally distinct endothelial phenotypes, consistent with the known concentration‐dependent effects of Adamts1 on angiogenesis.

### 2.4 *ADAMTS1* Overexpression Exacerbated Cardiac Dysfunction and Altered Scar Composition Post‐MI

Given that collagen matrix and ECM PGs are enriched during post‐MI remodeling and contribute to fibrotic scar formation (Figure 1B), we investigated the role of Adamts1, a major PG‐cleaving enzyme, in cardiac scar formation using our validated transgenic mouse model.

**Figure 3:**
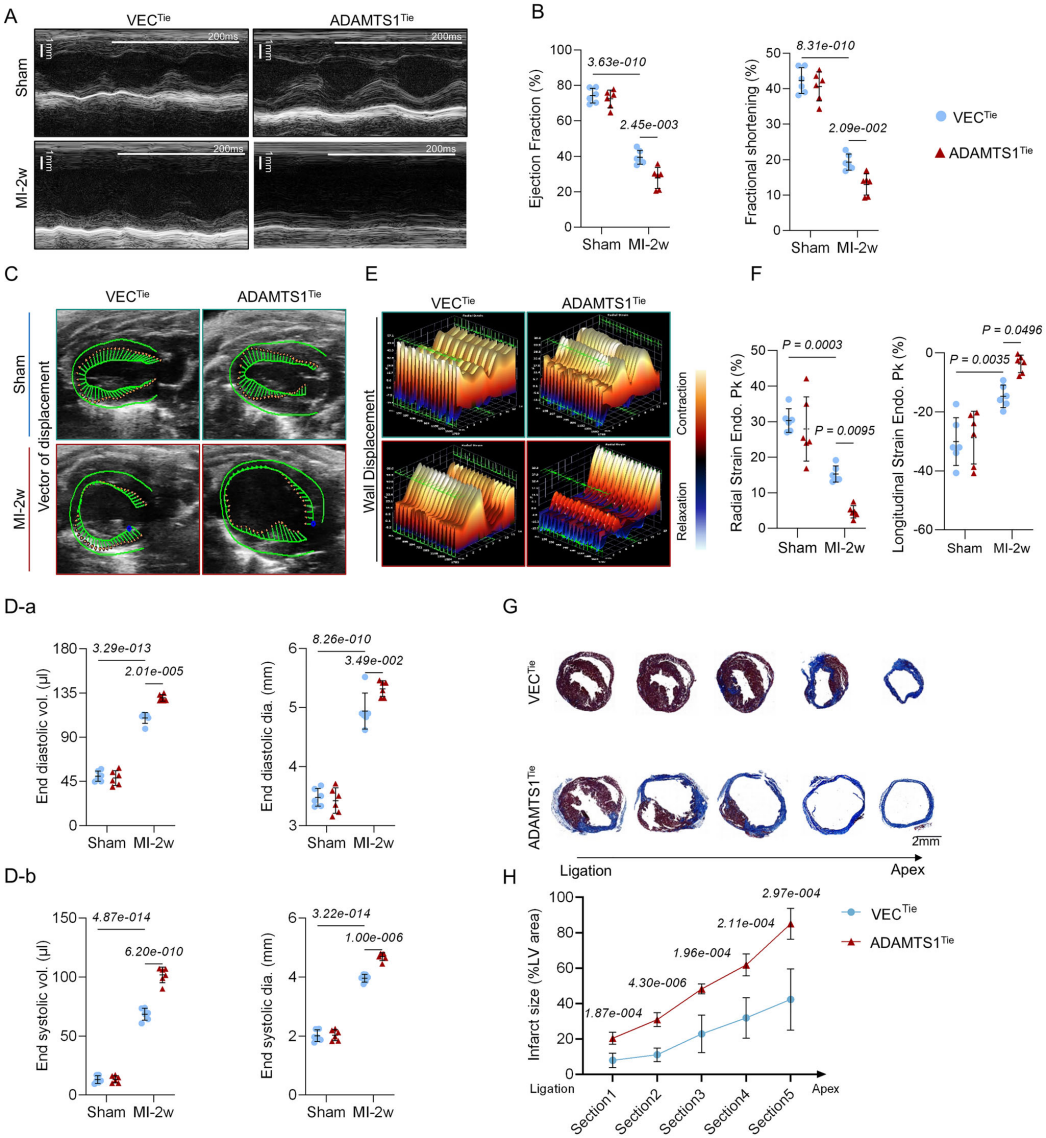
Overexpression of Adamts1 aggravates cardiac dysfunction and scar formation post‐MI. A,B) Representative M‐mode echocardiography images and quantitative analysis of cardiac function in VEC^Tie^ and ADAMTS1^Tie^ mice (*n* = 6 per group). C) Representative B‐mode echocardiographic images showing ventricular wall expansion and motion in ADAMTS1^Tie^ and VEC^Tie^ mice. D) Echocardiographic quantification of end‐systolic volume (ESV), end‐systolic diameter (ESD), stroke volume, and cardiac output at 2 weeks post‐MI in ADAMTS1^Tie^ and VEC^Tie^ mice (*n* = 6 per group). E) Representative 3D speckle tracking images demonstrating endocardial wall displacement. F) Quantitative analysis of radial strain and longitudinal strain in endocardial motility (*n* = 6 per group). G,H) Masson's trichrome staining and quantitative analysis of infarct area (IA) from ligation site to apex in VEC^Tie^ and ADAMTS1^Tie^ mice (*n* = 5 per group). *t* (Section 1) = 5.743, *t* (Section 2) = 8.963, *t* (Section 3) = 5.710, *t* (Section 4) = 5.655, *t* (Section 5) = 5.410. Statistical analysis was performed using multiple unpaired *t* tests with two‐stage step‐up method of Benjamini, Krieger, and Yekutieli correction H, and two‐way ANOVA followed by Bonferroni post hoc test in B, D, and F. Data are presented as mean ± SD. Adamts1, a disintegrin and metalloproteinase with thrombospondin motif 1; VEC, vector; MI, myocardial infarction; vol, volume; dia, diameter; Endo, endocardium; Pk, peak; LV, left ventricular.

ADAMTS1 overexpression in healthy mice (Sham + ADAMTS1^Tie^) did not affect cardiac function, possibly due to limited substrate availability in normal cardiac tissue. However, 2 weeks post‐ischemic injury, the ADAMTS1^Tie^ mice exhibited significantly greater cardiac dysfunction, evidenced by decreased systolic function (ejection fraction [EF] and fractional shortening [FS]) and consistent ventricular dilation (**Figure** **3**A–D). Speckle tracking analysis further confirmed attenuated ventricular wall motion and reduced radial/longitudinal strain, indicating diminished overall cardiac contractility (Figure 3E,F). Moreover, Masson's trichrome staining revealed significantly larger scar sizes in the ADAMTS1^Tie^ mice post‐MI (Figure 3G,H). These findings demonstrate that Adamts1 overexpression significantly impairs both cardiac function and increases scar burden post‐MI.

**Figure 4:**
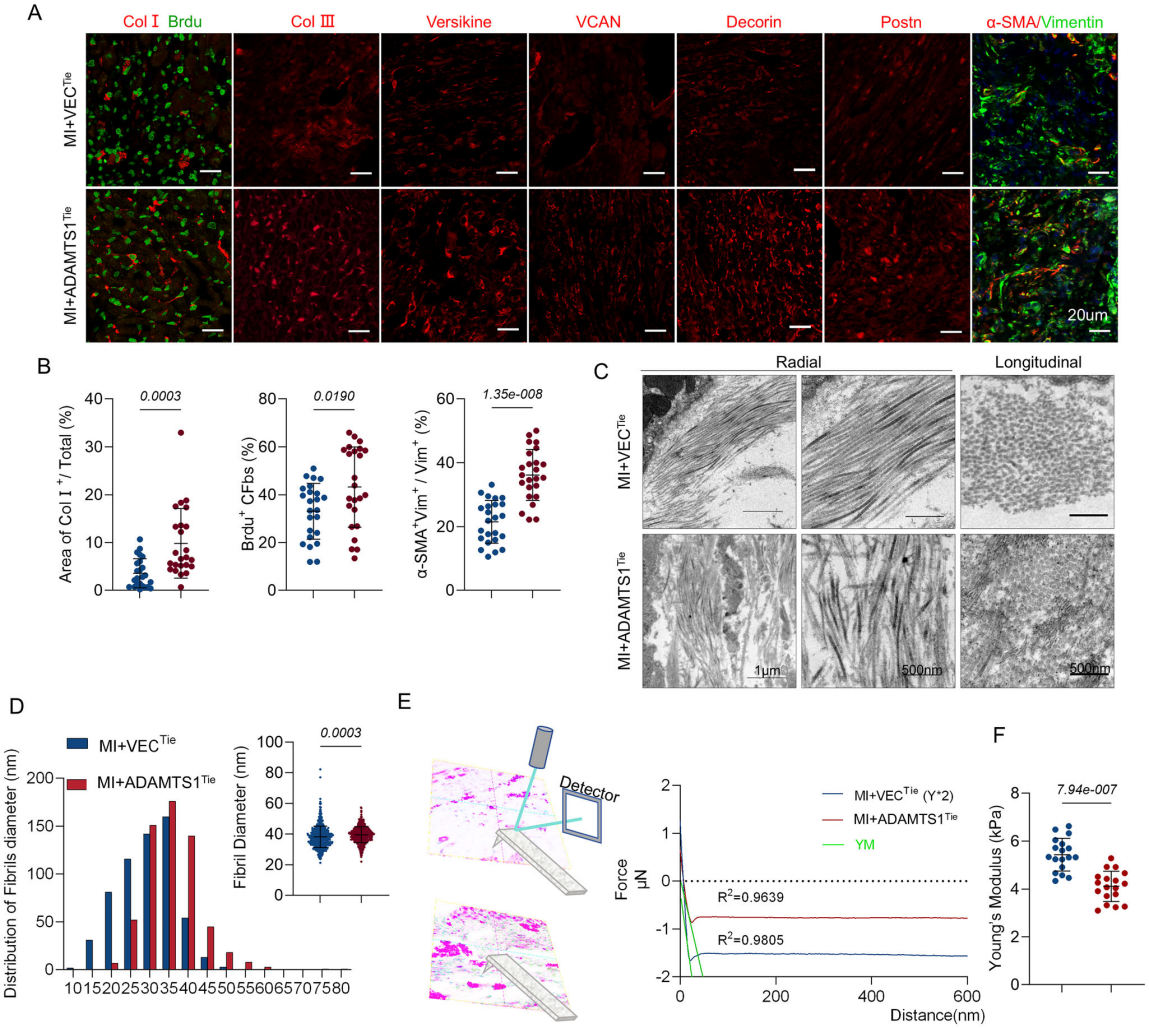
Overexpression of Adamts1 increases the accumulation of extracellular matrix (ECM) and tunes the mechanical structure of infarct scar. A) Immunofluorescence (IF) staining showing cardiac fibroblast activation and proliferation, and ECM accumulation in infarct scar from ADAMTS1^Tie^ and VEC^Tie^ mice. B) Quantitative analysis of collagen I area, BrdU^+^ cardiac fibroblasts, and α‐SMA^+^ area (*n* = 6, 4 fields per replicate). Statistical significance; *t* (ColI area) = 3.887, *t* (Brdu^+^ CFs) = 2.432; *t* (α‐SMA) = 6.886. C) Representative transmission electron microscopy (TEM) images showing scar fiber organization in ADAMTS1^Tie^ and VEC^Tie^ mice. D) Histogram showing distribution of collagen fiber diameters and quantitative analysis of fiber diameter (*n* = 3 per group, *t* = 3.673). E) Schematic diagram of Young's modulus detection by atomic force microscopy (AFM) and representative force–distance curves. F) Quantitative analysis of Young's modulus in scar tissue (*n* = 3 per group, *t* = 6.027). Statistical analysis was performed using unpaired t tests. Data are presented as the mean ± SD. Adamts1, a disintegrin and metalloproteinase with thrombospondin motif 1; Col, collagen; VCAN, versican; YM, Young's modulus.

To assess Adamts1's impact on scar composition, we examined collagen I/III levels, PGs (decorin, versican, and versikine), and cardiac fibroblast activation. Matrix protein levels were elevated, correlating with increased CFs proliferation and activation (**Figure** **4**A,B). Furthermore, transmission electron microscopy (TEM) revealed disorganized scar fibers with larger diameter distributions in the ADAMTS1^Tie^ mice (Figure 4C,D). Additionally, atomic force microscopy (AFM) measurements indicated a lower elastic modulus in the ADAMTS1^Tie^ mice than in the VEC^Tie^ group, indicating altered scar tissue mechanics (Figure 4E,F). Collectively, these data indicate that Adamts1 overexpression not only increases ECM deposition but also fundamentally alters the structural and mechanical properties of cardiac scars.

Despite significant functional impairments observed in isolated ECs, including reduced angiogenic capacity and impaired migration (Figure S4, Supporting Information), *ADAMTS1* overexpression resulted in only modest, non‐significant changes in capillary and arteriolar density in vivo (Figure S5A–C, Supporting Information). This disconnect between in vitro endothelial dysfunction and minimal in vivo vascular remodeling suggests that Adamts1's primary pathological effects in the post‐MI heart may operate through alternative mechanisms beyond direct vascular modulation.

Long‐term survival analysis showed that ADAMTS1^Tie^ mice had significantly reduced 28‐day survival compared to VEC^Tie^ controls. Cardiac rupture incidence was markedly higher in ADAMTS1^Tie^ mice (12/26, 46.2%) compared to VEC^Tie^ mice (3/22, 13.6%) (Figure S6A–C, Supporting Information).

### 2.5 Adamts1 Deficiency Improved Cardiac Function and Minimized Scar Size Post‐MI

**Figure 5:**
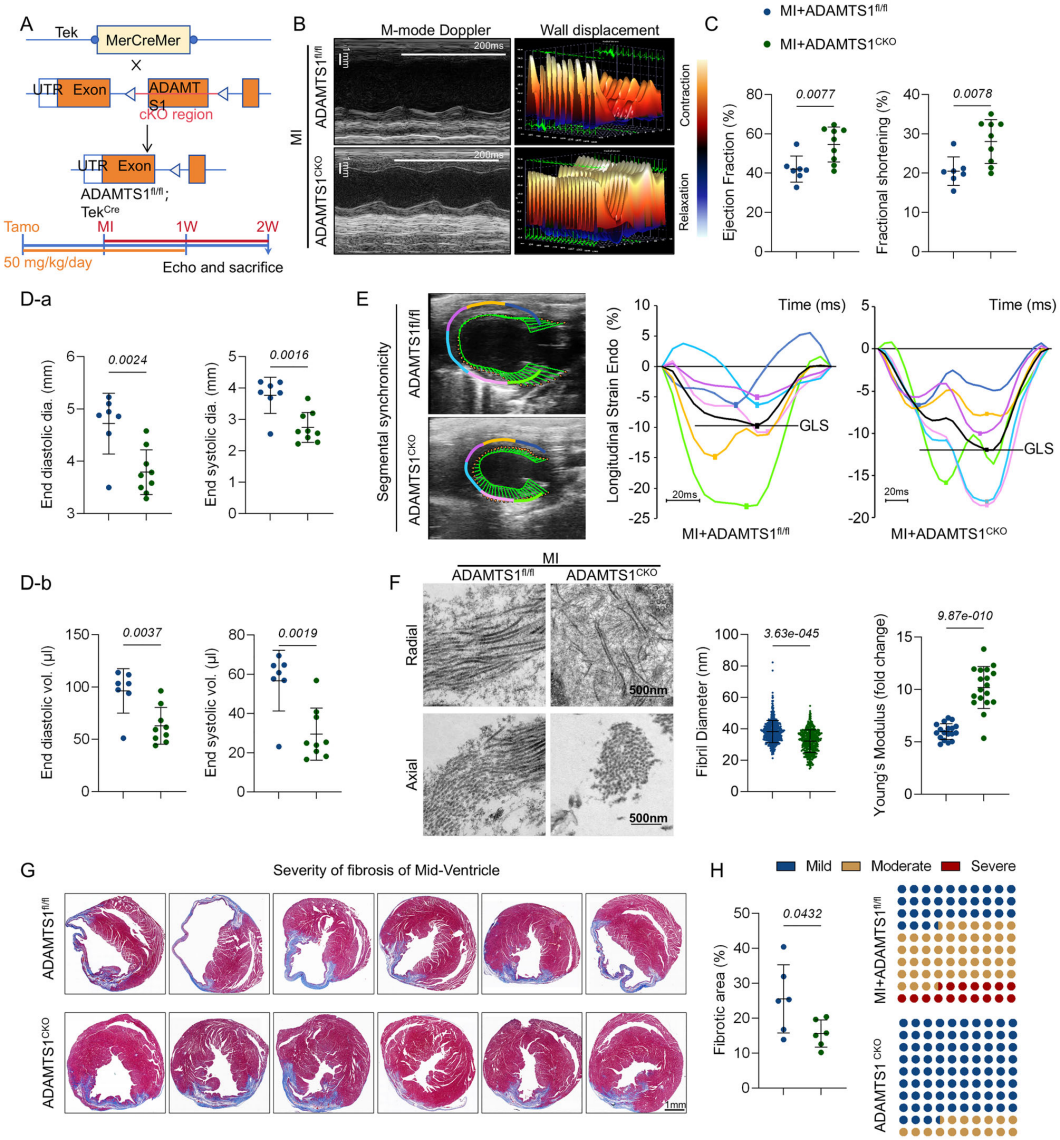
Adamts1 deficiency improves cardiac function and reduces scar formation Post‐MI. A) Schematic illustration of Adamts1 conditional knockout (CKO) mouse generation and experimental timeline. Tamoxifen (50 mg kg^−1^) was administered 1 week before and after MI surgery. B) Representative M‐mode echocardiographic images and 3D speckle tracking analysis showing wall displacement in ADAMTS1^CKO^ and ADAMTS1^fl/fl^ mice at 2 weeks post‐MI. C) Quantitative analysis of cardiac systolic function parameters including ejection fraction (EF, *t* = 3.107) and fractional shortening (FS, *t* = 3.100) in ADAMTS1^CKO^ (*n* = 9) and ADAMTS1^fl/fl^ mice (*n* = 7). D) Echocardiographic measurements of (a) end‐diastolic diameter (EDD, *t* = 3.689) and end‐systolic diameter (ESD, *t* = 3.893), and (b) end‐diastolic volume (EDV, *t* = 3.476) and end‐systolic volume (ESV, *t* = 3.803) in ADAMTS1^CKO^ and ADAMTS1^fl/fl^ mice. E) Representative B‐mode echocardiographic images showing ventricular wall expansion and regional myocardial motion in ADAMTS1^CKO^ and ADAMTS1^fl/fl^ mice. F) Transmission electron microscopy (TEM) images of scar tissue and quantitative analysis of fibril diameter and Young's modulus ADAMTS1^CKO^ and ADAMTS1^fl/fl^ mice (*n* = 3 per group; fibril diameter: *t* = 14.71; Young's modulus: *t* = 8.337). Scale bars = 500 nm. G) Representative Masson's trichrome staining of cardiac cross‐sections from apex to base showing fibrotic areas (blue) in ADAMTS1^CKO^ and ADAMTS1^fl/fl^ mice. Scale bars = 1 mm. H) Quantitative analysis of fibrosis severity classified as mild, moderate, and severe, with dot plot showing distribution of fibrotic area percentage in ADAMTS1^CKO^ and ADAMTS1^fl/fl^ mice (*n* = 6 per group; *t* = 2.314). Statistical analyses were performed using unpaired *t*‐test. Data are presented as mean ± SD.

To further elucidate the biological effects of *ADAMTS1*, we generated endothelium‐specific *ADAMTS1* conditional knockout (ADAMTS1^CKO^) mice. Littermate ADAMTS1^fl/fl^ mice without Tek‐iCre transgene were assigned as control animals. Both groups received identical tamoxifen treatment (50 mg kg^−1^ day^−1^) 1 week before and after MI surgery (**Figure** **5**A).

Knockout efficiency was validated through multiple approaches. IF analysis confirmed markedly reduced Adamts1 protein expression specifically in CD31^+^ ECs in ADAMTS1^CKO^ mice (Figure S7A, Supporting Information). qRT‐PCR analysis of isolated cardiac cell populations demonstrated significantly reduced *ADAMTS1* mRNA levels in ECs from ADAMTS1^CKO^ mice, while CFs and cardiomyocytes showed no significant changes (Figure S7B, Supporting Information). Western blot analysis of isolated cell populations further validated the endothelial‐specific knockout efficiency (Figure S7C, Supporting Information).

Two weeks post‐MI, echocardiography revealed improved systolic function, reduced ventricular dilation, and enhanced ventricular wall motion in the ADAMTS1^CKO^ mice compared to the littermate controls (Figure 5B–E). Further analysis of scar structure and mechanical properties demonstrated that the matrix fibers in ADAMTS1‐deficient mice were narrower and exhibited a higher elastic modulus (Figure 5F). Additionally, quantification of the fibrotic area, with moderate fibrosis defined as 20‐40% of the affected myocardium, indicated that *ADAMTS1* deletion significantly reduced both the proportion of severe fibrosis and overall scar size (Figure 5G,H).

Extended survival monitoring demonstrated no significant difference in 28‐day survival between ADAMTS1^CKO^ and ADAMTS1^fl/fl^ mice, with similar cardiac rupture incidence rates (11.1% versus 12.5%) (Figure S8A,B, Supporting Information). These findings demonstrate that Adamts1 deficiency improves cardiac function and reduces pathological scar formation without compromising cardiac structural integrity.

### 2.6 Adamts1 Regulation of Post‐MI Scar Formation May Be Mediated by ITGα8 Signaling

**Figure 6:**
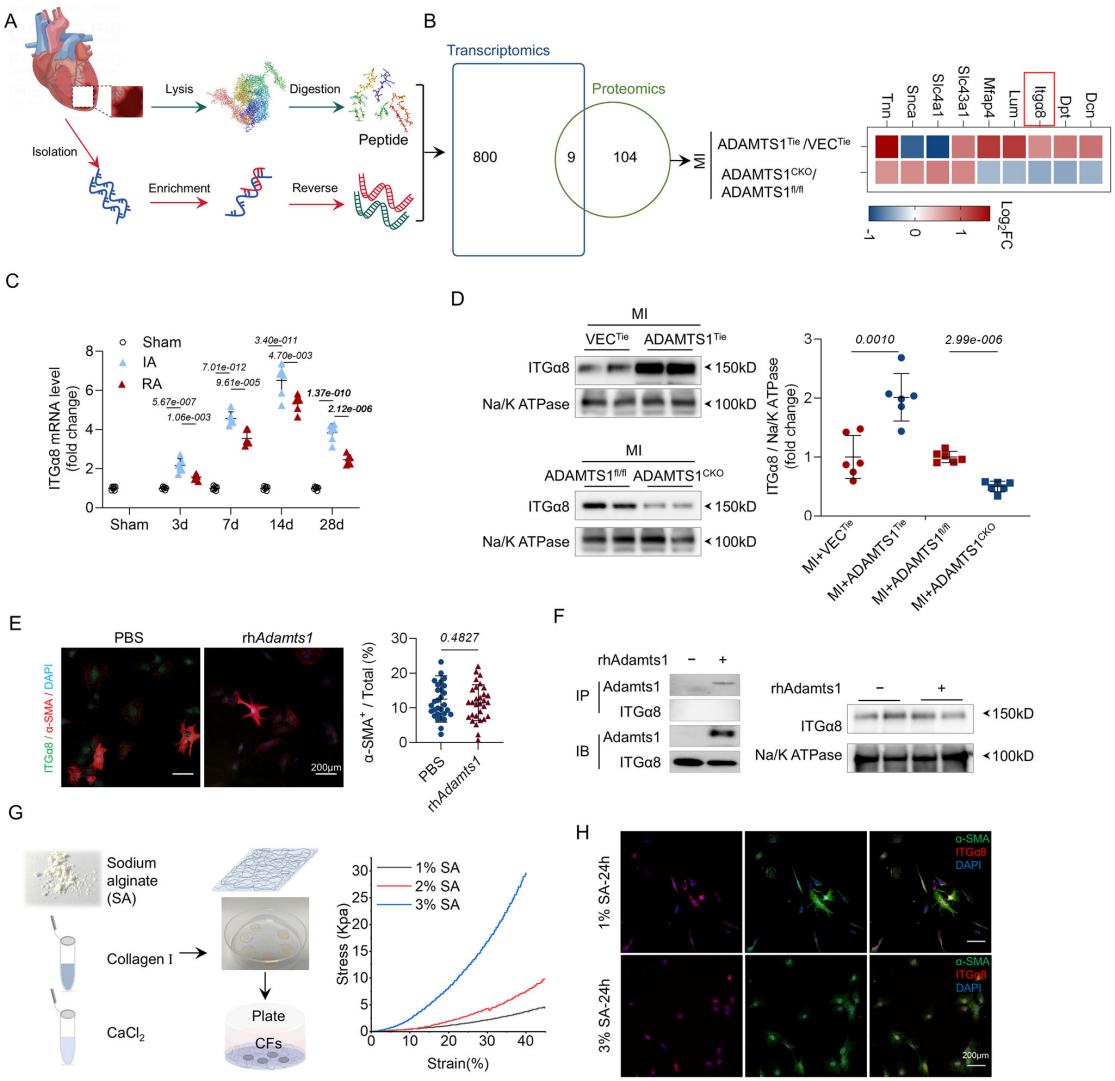
Mechanical activation of ITGα8 by Adamts1 regulates scar formation. A) Schematic illustration of experimental workflow for combined transcriptome and proteome analysis, showing tissue isolation, processing, and multi‐omics analysis pipeline. B) Venn diagram showing overlap between transcriptome (809 genes) and proteome (113 proteins) datasets, and heatmap displaying opposing expression patterns of selected genes in ADAMTS1^Tie^ versus ADAMTS1^CKO^ mice (*n* = 5 and 3). C) Transcript level of *ITGa8* at different time points post‐MI (*n* = 6 per group). 3 days: F (2,15) = 40.74, *p* = 8.66e‐007, 7 days: F (2, 15) = 221.1, *p* = 7.41e‐012, 14 days: F (2, 15) = 186.3, *p* = 2.56e‐011. 28 days: F (2, 15) = 138.0, *p* = 2.19e‐010. D) Western blot analysis of ITGα8 and Adamts1 protein expression in ADAMTS1^Tie^ and ADAMTS1^CKO^ mice (*n* = 6); *t* (Adamts1^Tie^) = 4.562, *t* (ADAMTS1^CKO^) = 9.329. E) Immunofluorescence (IF) staining of ITGα8 in cardiac fibroblasts treated with recombinant human Adamts1 protein (*n* = 5, at least six fields per replicate, *t* = 0.7064). F) Co‐immunoprecipitation analysis examining protein interactions between Adamts1 and ITGα8. G) Schematic diagram of tunable stiffness sodium alginate (SA) hydrogel preparation and mechanical characterization. H) IF staining of cardiac fibroblasts cultured on 1% and 3% SA hydrogels. Statistical analysis was performed using unpaired *t* tests (C and D). Data are presented as the mean ± SD. Adamts1, a disintegrin and metalloproteinase with thrombospondin motif 1; VEC, vector; CKO, conditional knockout; Tnn, tenascin n; Snca, synuclein α; Slc4a1, solute carrier family 4 member 1; Slc43a1, solute carrier family 43 member 1; Mfap4, microfibril associated protein 4; Lum, lumican; ITGα8, integrin subunit α8; Dpt, dermatopontin; rhAdamts1, recombinant human Adamts1 protein.

CFs activation and proliferation play a crucial role in scar maturation.^[^ ^22^ ^]^ Moreover, mechanical forces are considered key mediators in promoting fibroblast‐to‐myofibroblast differentiation.^[^ ^23^ ^]^ To identify the molecular mechanisms driving Adamts1‐mediated scar formation, we employed a comprehensive multi‐omics approach combining transcriptomics and proteomics (**Figure** **6**A). Subsequently, integrated gene pathway analysis (Metascape) revealed significant alterations in the pathways associated with ECM organization, ECM‐PGs, ITG cell surface interactions, and ECM‐receptor interactions (Figure S9A, Supporting Information). Furthermore, gene classification analysis demonstrated coordinated upregulation of collagen proteins, ECM regulators, cell adhesion molecules, signaling factors, and PGs following Adamts1 overexpression (Figure S9B, Supporting Information).

Comparative analysis between ADAMTS1^Tie^ transcriptome and ADAMTS1^CKO^ proteome datasets identified genes and proteins exhibiting opposing expression patterns, with ITGα8 emerging as a prime candidate (Figure 6B). The ITG family, acting as cell surface mechanosensitive receptors, is influenced by the mechanical environment of the ECM.^[^ ^24^, ^25^ ^]^ This mechanical environment may trigger ITG receptor activation, suggesting that ITG receptors could potentially mediate Adamts1‐regulated scar formation. A comparative analysis of transcriptome profiles from ADAMTS1^Tie^ mice and proteome profiles from ADAMTS1^CKO^ mice revealed opposing expression patterns for ITGα8, tenascin‐N (TNN), dermatopontin (DPT), lumican (LUM), and decorin (Figure 6B, Tables S3 and S4, Supporting Information). These ECM components regulate intercellular adhesion via ITGs and mediate ITG‐dependent cell migration.^[^ ^26^ ^]^ ITGα8, a member of the ITG family, is involved in various cellular processes, including cell adhesion, ECM synthesis, and CF activation.^[^ ^27^, ^28^ ^]^ Time‐course analysis revealed sustained *ITGα8* upregulation post‐MI, with peak expression at 14 days persisting through 28 days (Figure 6C), closely paralleling the temporal dynamics observed for *ADAMTS1* expression. Broader analysis of the integrin family showed that ITGα2, ITGα4, and ITGα8 were consistently upregulated across all experimental groups following MI (Figure S9C, Supporting Information). Protein‐level validation confirmed that ITGα8 expression directly correlated with Adamts1 levels, increasing in ADAMTS1^Tie^ mice while decreasing in ADAMTS1^CKO^ mice (Figure 6D).

To assess integrin family specificity, we examined all major cardiac integrin subunits. While ADAMTS1 overexpression elevated ITGα2, ITGα4, ITGα8, and ITGα11 (Figure S9D, Supporting Information), Adamts1 deletion selectively reduced only ITGα8 expression, leaving other integrin subunits unchanged (Figure S9E, Supporting Information). Additionally, although TGF‐β1 and mechanical tension are considered key signaling mediators responsible for promoting CFs activation,^[^ ^29^ ^]^ our data indicated that the TGF‐β family was not affected by Adamts1 deficiency, thus excluding its potential role in *ADAMTS1*‐mediated scar regulation (Figure S9F, Supporting Information). These findings suggest that ITGα8 may specifically be involved in *ADAMTS1*‐regulated scar formation.

### 2.7 Adamts1 Did Not Interact Directly with ITGα8

To elucidate the activation mechanism between Adamts1 and ITGα8, ZDOCK was initially employed to simulate their interactions. Consequently, it predicted potential polar bond formation between their 3D protein structures (ADAMTS1 ID: Q9UHI8; ITGα8 ID: P53708) (Figure S10A, Supporting Information). However, experimental validation yielded contrasting results. Direct treatment of CFs with recombinant human Adamts1 failed to alter any integrin subtype expression or increase α‐SMA^+^ cell proportions (Figure S10B,C, Supporting Information). Consistently, IF analysis confirmed that recombinant Adamts1 did not activate ITGα8 in cultured fibroblasts (Figure 6E).

Co‐immunoprecipitation (Co‐IP) experiments definitively ruled out direct protein interactions between Adamts1 and ITGα8 (Figure 6F). We also explored alternative indirect pathways, examining whether Adamts1 might influence ITGα8 through the VCAN‐hyaluronic acid (HA) network or CD44 receptor signaling. The VCAN‐HA supramolecular network is known to store significant amounts of TGF‐β precursors that promote fibroblast activation.^[^ ^30^ ^]^ Additionally, the primary HA receptor, CD44, plays a crucial role in fibroblast migration.^[^ ^31^ ^]^ Analysis revealed no significant changes in HA accumulation or CD44 activation between ADAMTS1^Tie^ and control mice (Figure S10D,E, Supporting Information). These comprehensive findings demonstrate that Adamts1 regulates ITGα8 through indirect mechanisms rather than direct molecular interactions.

### 2.8 Mechanotransduction Mediates ITGα8 Activation Through Altered ECM Stiffness

Having excluded direct interactions, we hypothesized that mechanical cues might mediate ITGα8 activation. We engineered sodium alginate (SA) hydrogels with tunable mechanical properties to test this hypothesis (Figure 6G). Mechanical characterization revealed that 1% and 2% hydrogels recapitulated the softer mechanical environment measured in ADAMTS1^Tie^ scars, while 3% hydrogels mimicked the stiffer properties of ADAMTS1^CKO^ scar tissue (Figure S11A,B, Supporting Information). This design enabled physiologically relevant testing of our mechanotransduction hypothesis.

Fibroblast culture experiments demonstrated striking mechanical sensitivity. Cells cultured on softer substrates (1% SA) exhibited markedly enhanced myofibroblast differentiation compared to those on stiffer substrates (3% SA) (Figure 6H). Quantitative analysis confirmed significantly higher α‐SMA^+^ cell proportions on compliant versus rigid substrates (Figure S11C,D, Supporting Information). These results establish that mechanotransduction—mediated by ECM stiffness rather than direct protein binding—constitutes the primary mechanism through which Adamts1 regulates ITGα8‐dependent cardiac fibroblast activation and pathological scar formation.

### 2.9 ITGα8 Deficiency Reduced CF Proliferation, Activation, and Contractility

Since myofibroblast‐mediated ECM protein synthesis and contractile forces are crucial determinants of scar formation and size,^[^ ^32^ ^]^ we investigated the functional impact of ITGα8 deficiency on cardiac fibroblast activity. We generated ITGα8 conditional knockout (ITGα8^CKO^) mice by cross‐breeding ITGα8^fl/fl^ mice with fibroblast‐specific Col1α2‐CreER mice.

To confirm the efficiency and specificity of ITGα8 deletion, we performed comprehensive validation using multiple approaches. IF analysis of cardiac tissue sections demonstrated markedly reduced ITGα8 expression specifically in α‐SMA^+^ CFs in ITGα8^CKO^ mice compared to ITGα8^fl/fl^ controls (Figure S12A, Supporting Information). Cell‐type‐specific qRT‐PCR analysis of isolated cardiac cell populations revealed that *ITGα8* mRNA levels were significantly reduced in CFs from ITGα8^CKO^ mice, while ECs and cardiomyocytes showed no significant changes (Figure S12B, Supporting Information). Western blot analysis of isolated cell populations further confirmed fibroblast‐specific ITGα8 protein reduction, with preserved expression of cell‐type specific markers (Figure S12C, Supporting Information). These findings confirm the fibroblast specificity of our genetic modification at both transcriptional and protein levels.

**Figure 7:**
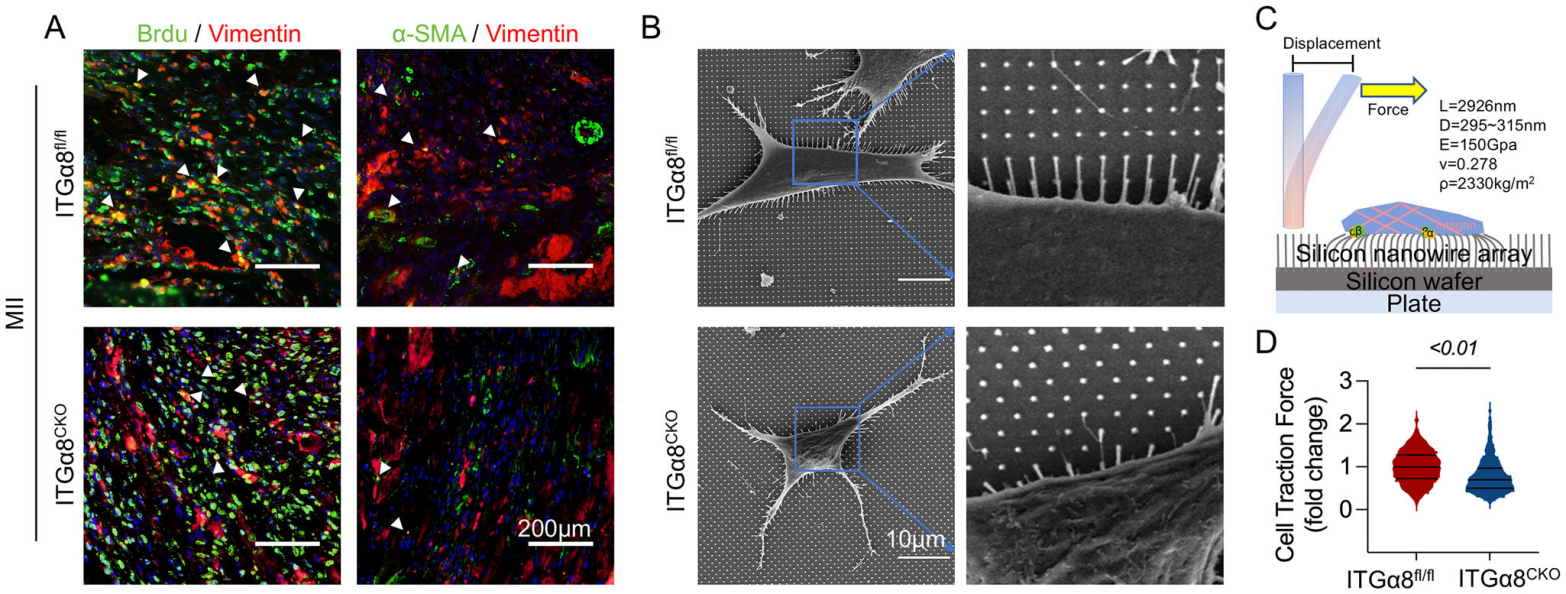
ITGα8 deficiency attenuates cardiac fibroblast activation, proliferation, and contractility. A) Representative immunofluorescence (IF) images of cardiac tissue sections from ITGα8^fl/fl^ and ITGα8^CKO^ mice at 2 weeks post‐MI. Left panels show BrdU (green, proliferation marker) and vimentin (red, fibroblast marker) co‐staining. Right panels show α‐SMA (red, myofibroblast activation marker) and vimentin (green) co‐staining. DAPI (blue) indicates nuclei. Scale bars = 200 µm. B) Representative scanning electron microscopy (SEM) images of cardiac fibroblasts cultured on silicon nanopillar (Si‐NP) arrays showing cellular morphology and nanopillar deflection in ITGα8^fl/fl^ and ITGα8^CKO^ groups. Scale bars= 10 µm. C) Schematic illustration of the silicon nanopillar array system for measuring cellular contractile forces. Individual nanopillars deflect in response to cellular traction forces, allowing quantitative assessment of cell contractility. D) Quantitative analysis of cardiac fibroblast contractile force measured by nanopillar deflection in ITGα8^fl/fl^ and ITGα8^CKO^ groups (*n* = 2, at least 10 fields of view per biological replicate; *t* = 8.617). Statistical analysis was performed using unpaired *t*‐test. Data are presented as mean ± SD.

Two weeks post‐MI, IF analysis revealed that ITGα8^CKO^ mice exhibited significantly reduced numbers of both proliferating (BrdU^+^) and activated (α‐SMA^+^) CFs compared to ITGα8^fl/fl^ controls (**Figure** **7**A). To assess the impact on fibroblast contractile capacity, we employed silicon nanopillar array technology to quantitatively measure cellular traction forces. Scanning electron microscopy (SEM) revealed distinct morphological differences between CFs isolated from ITGα8^CKO^ and ITGα8^fl/f^ mice, with reduced nanopillar deflection indicating decreased contractile activity in ITGα8‐deficient cells (Figure 7B). The silicon nanopillar system enables precise measurement of cellular contractile forces through quantitative analysis of nanopillar displacement (Figure 7C). Quantitative analysis demonstrated that ITGα8^CKO^ CFs exhibited significantly reduced contractile force compared to controls (Figure 7D).

These comprehensive results establish that ITGα8 plays essential roles in cardiac fibroblast activation, proliferation, and contractile function, demonstrating its critical importance in regulating scar matrix formation and mechanical properties post‐MI.

### 2.10 ITGα8 Deficiency Improved Post‐MI Cardiac Dysfunction

To clarify the role of ITGα8 in cardiac function regulation, ITGα8^fl/fl^ and ITGα8^CKO^ mice underwent MI surgery to assess the impact of *ITGα8* deletion on cardiac function. Echocardiographic analysis at 2 weeks post‐MI revealed that ITGα8^CKO^ mice exhibited significantly improved cardiac performance compared to ITGα8^fl/fl^ controls. Representative M‐mode, B‐mode, and 3D speckle tracking images demonstrated more preserved ventricular wall motion and cardiac morphology in ITGα8^CKO^ mice (Figure S13A, Supporting Information). Quantitative analysis of systolic function parameters showed that ITGα8^CKO^ mice maintained significantly higher EF and FS compared to controls (Figure S13B, Supporting Information). Assessment of ventricular remodeling parameters revealed that ITGα8 deletion attenuated adverse cardiac remodeling. ITGα8^CKO^ mice demonstrated smaller ventricular dimensions, with reduced end‐diastolic and end‐systolic diameters (Figure S13C, Supporting Information). Volumetric analysis further confirmed these findings, showing decreased end‐diastolic volumes (EDVs) and end‐systolic volumes (ESVs) in ITGα8^CKO^ mice (Figure S13D, Supporting Information). These parameters collectively indicate that ITGα8 deletion preserves cardiac contractile function and limits pathological ventricular enlargement following MI.

Long‐term monitoring demonstrated that ITGα8 deletion maintained an excellent safety profile. Survival analysis over 28 days showed comparable survival rates between all experimental groups, with no significant differences between ITGα8^CKO^ and control mice (Figure S13E, Supporting Information). Importantly, cardiac rupture incidence remained similar between MI+ITGα8^fl/fl^ and MI+ITGα8^CKO^ groups, indicating that ITGα8 deletion does not compromise cardiac structural integrity (Figure S13F, Supporting Information). These findings establish that ITGα8 deletion provides functional benefits without increasing the risk of adverse cardiac events, suggesting that targeting ITGα8 represents a safe therapeutic approach for improving post‐MI outcomes.

### 2.11 ITGα8 Deficiency Blocked Adamts1‐Mediated Cardiac Dysfunction and Scar Formation

To determine whether ITGα8 mediates *ADAMTS1* overexpression‐induced cardiac dysfunction, we generated mice with both cardiac fibroblast‐specific ITGα8 deletion and EC‐specific *ADAMTS1* overexpression. This experimental design allowed us to test whether ITGα8 deficiency could rescue the detrimental effects of *ADAMTS1* overexpression on cardiac function and scar formation.

**Figure 8:**
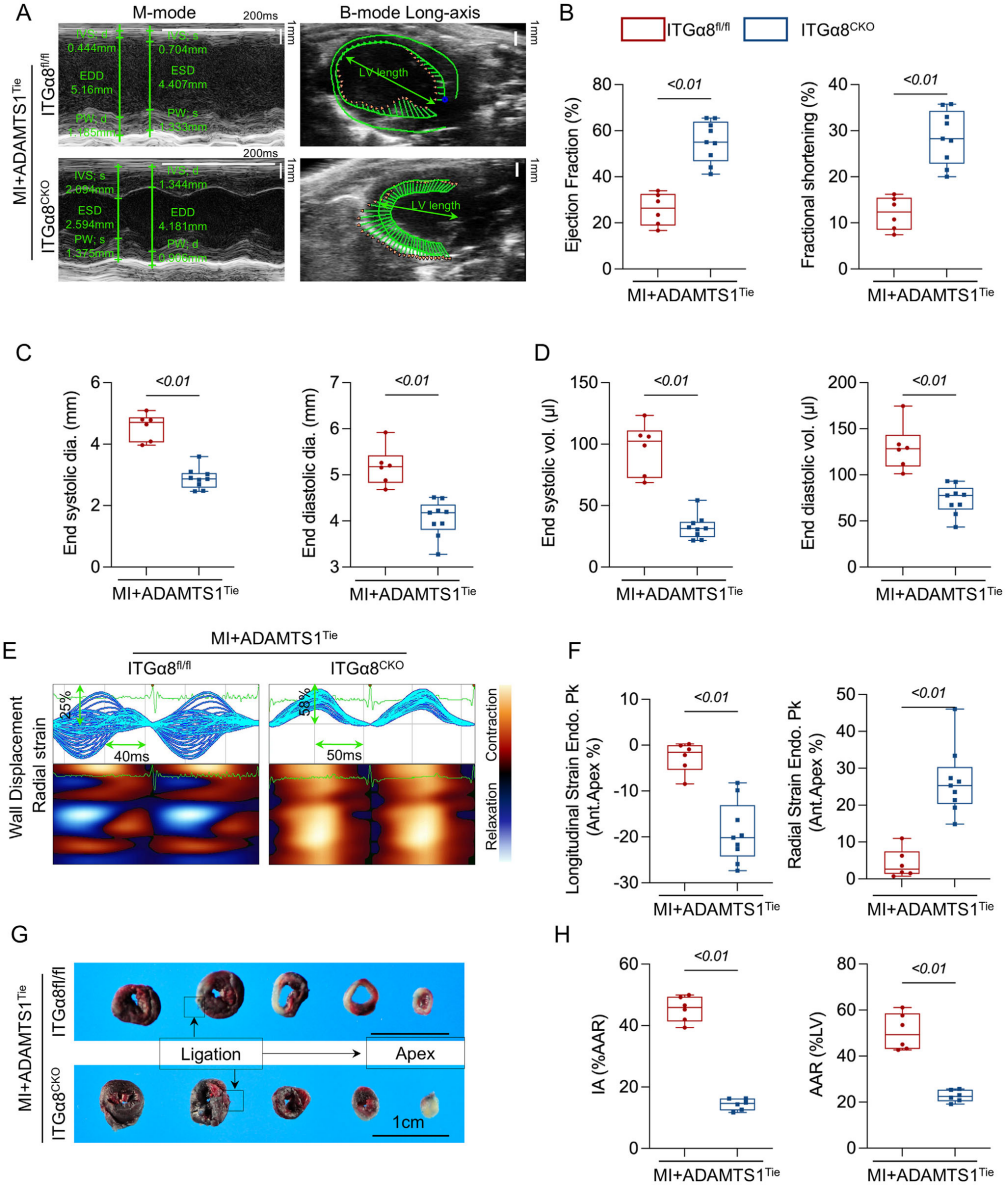
ITGα8 deficiency rescues cardiac dysfunction and reduces infarct size in Adamts1 overexpression mice post‐myocardial infarction. A) Representative M‐mode and B‐mode long‐axis echocardiographic images showing cardiac function in MI+ADAMTS1^Tie^ mice with ITGα8^fl/fl^ and ITGα8^CKO^ backgrounds at 2 weeks post‐MI. B) Quantitative analysis of cardiac systolic function parameters including ejection fraction (EF, *t* = 6.768) and fractional shortening (FS, *t* = 6.175) in MI+Adamts1^Tie^ mice with ITGα8^fl/fl^ and ITGα8^CKO^ backgrounds (*n* = 6 for ITGα8^fl/fl^, *n* = 9 for ITGα8^CKO^). C) Echocardiographic measurements of end‐diastolic diameter (EDD, *t* = 5.313) and end‐systolic diameter (ESD, *t* = 8.304) in the two experimental groups. D) Quantitative analysis of end‐diastolic volume (EDV, *t* = 5.359) and end‐systolic volume (ESV, *t* = 7.977) in ITGα8^fl/fl^ and ITGα8^CKO^ mice with Adamts1 overexpression. E) Representative 3D speckle tracking echocardiographic images showing wall displacement patterns in ITGα8^fl/fl^ and ITGα8^CKO^ mice with Adamts1 overexpression. F) Quantitative analysis of longitudinal strain at end‐apex (*t* = 5.623) and radial strain at end‐apex (*t* = 5.629) in the two experimental groups. G) Representative Evans blue and 2,3,5‐triphenyltetrazolium chloride (TTC) staining of cardiac sections from ligation site to apex. Non‐ischemic myocardium appears blue, infarct area (IA) appears pale/white, and area at risk (AAR) appears red. Scale bar = 1 cm. H) Quantitative analysis of IA to area at risk ratio (IA/AAR) and area at risk to left ventricle ratio (AAR/LV) in ITGα8^fl/fl^ and ITGα8^CKO^ mice with Adamts1 overexpression (*n* = 6 per group). Statistical analyses were performed using unpaired *t*‐test. Data are presented as mean ± SD.

Echocardiographic analysis revealed that ITGα8^CKO^ mice maintained significantly better cardiac function compared to ITGα8^fl/fl^ controls, even under conditions of *ADAMTS1* overexpression. Representative M‐mode and B‐mode echocardiographic images demonstrated preserved ventricular wall motion and cardiac morphology in ITGα8^CKO^ mice (**Figure** **8**A). Quantitative analysis of systolic function parameters showed that ITGα8 deletion dramatically improved both EF and FS compared to controls (Figure 8B). Assessment of cardiac remodeling parameters revealed that ITGα8^CKO^ mice exhibited reduced ventricular dilation across all measured dimensions. Both end‐diastolic and end‐systolic diameters were significantly smaller in ITGα8^CKO^ mice (Figure 8C), and this improvement extended to volumetric measurements, with reduced EDVs and ESVs (Figure 8D). Advanced cardiac imaging using 3D speckle tracking demonstrated enhanced ventricular wall motion patterns in ITGα8^CKO^ mice (Figure 8E), with improved regional strain parameters including both longitudinal and radial strain (Figure 8F).

To assess the impact on myocardial injury, we performed TTC staining to evaluate infarct size. Representative cardiac sections revealed markedly reduced IAs in ITGα8^CKO^ mice compared to ITGα8^fl/fl^ controls (Figure 8G). Quantitative analysis confirmed significant reductions in both IA to area at risk ratio and area at risk to left ventricle ratio in ITGα8^CKO^ mice (Figure 8H), indicating that ITGα8 deletion provides substantial cardioprotection even in the presence of *ADAMTS1* overexpression.

Long‐term survival analysis revealed the most striking benefit of ITGα8 deletion. Kaplan‐Meier survival curves demonstrated significantly improved 28‐day survival rates in MI+ADAMTS1^Tie^+ITGα8^CKO^ mice compared to MI+ADAMTS1^Tie^+ITGα8^fl/fl^ controls (Figure S14A, Supporting Information). Most remarkably, ITGα8 deletion dramatically reduced cardiac rupture incidence, providing near‐complete protection against this lethal complication (Figure S14B, Supporting Information).

These comprehensive findings establish that ITGα8 plays a crucial mediating role in *ADAMTS1* overexpression‐induced cardiac dysfunction and adverse remodeling. ITGα8 deletion effectively rescues cardiac functional impairment, reduces myocardial injury, and prevents life‐threatening complications, confirming that the Adamts1‐ITGα8 axis represents a critical pathway in post‐MI cardiac pathophysiology.

## 3 Discussion

Scar formation represents a critical determinant of structural and functional recovery following MI. Our study reveals a novel mechanistic pathway wherein EC‐derived Adamts1, significantly upregulated post‐MI, plays a pivotal role in regulating cardiac remodeling through mechanotransduction‐mediated activation of ITGα8 in CFs. We demonstrate that *ADAMTS1* overexpression exacerbates cardiac dysfunction by expanding scar area and altering scar mechanical properties, while ADAMTS1 deficiency provides cardioprotection. Mechanistically, our findings establish that Adamts1 regulates scar formation through PG cleavage, which reduces extracellular matrix stiffness and generates mechanical cues that specifically activate ITGα8 mechanosensing in CFs. This mechanotransduction pathway—rather than direct protein interactions—mediates cardiac fibroblast activation, proliferation, and excessive ECM synthesis. Importantly, ITGα8 deletion rescues ADAMTS1‐induced cardiac dysfunction and prevents adverse remodeling, confirming the essential role of this mechanosensitive pathway in post‐MI pathophysiology. These findings identify the Adamts1‐ITGα8 mechanotransduction axis as a novel therapeutic target for modulating post‐MI cardiac remodeling.

ADAMTS1 expression demonstrates peak levels at 14 days post‐MI, persisting through 28 days with predominant localization in the IA, establishing it as a sustained remodeling regulator. This expression pattern reflects transcriptional control by hypoxia‐inducible factor 1α (HIF‐1α) through direct promoter binding, creating an endothelial‐specific hypoxic response throughout scar formation.^[^ ^6^, ^33^ ^]^ Additional regulatory pathways include Krüppel‐like factor 6 (KLF6) in CFs^[^ ^34^ ^]^ and inflammatory cytokines TNF‐α and IL‐1β that enhance HIF‐1α stability through NF‐κB and MAPK signaling.^[^ ^35^ ^]^

A fundamental insight from our study concerns ADAMTS1's substrate‐dependent activity pattern, which reconciles apparent discrepancies between in vitro cellular dysfunction and limited in vivo effects. ADAMTS1 exhibits highly specific proteolytic activity toward PGs, cleaving versican at the Glu441‐Ala442 bond to release the bioactive G1‐DPEAAE fragment.^[^ ^36^, ^37^ ^]^ This catalytic activity requires accessible substrate presentation, which differs dramatically between healthy and pathological ECM environments. In healthy myocardium, PGs remain sequestered within organized ECM networks where cleavage sites are cryptic.^[^ ^38^, ^39^ ^]^ This explains why ADAMTS1 overexpression in healthy mice produced no cardiac effects—the enzyme lacks appropriate substrate availability for meaningful catalytic activity. Conversely, the post‐MI environment creates optimal conditions through extensive PG accumulation and inflammatory‐mediated ECM disorganization that exposes cryptic cleavage sites,^[^ ^40^, ^41^ ^]^ positioning ADAMTS1 as both a sensor and modifier of pathological ECM states rather than a constitutive matrix‐degrading enzyme. The substrate‐dependent activity of ADAMTS1 provides one explanation for why overexpression affects only post‐MI hearts. However, we cannot exclude the possibility that other factors, such as inflammatory mediators or tissue hypoxia, may also contribute to this specificity.

A notable finding was the discrepancy between in vitro endothelial dysfunction and relatively limited in vivo vascular alterations following ADAMTS1 overexpression. This disconnect reflects sophisticated compensatory mechanisms within the intact cardiovascular system, where redundant angiogenic pathways provide critical buffering against single‐factor perturbations. The cardiac microenvironment maintains multiple parallel pro‐angiogenic circuits, including VEGF/VEGFR2, FGF/FGFR, and PDGF/PDGFR signaling axes. These pathways converge on common downstream targets to sustain endothelial survival and proliferation.^[^ ^42^ ^]^ Furthermore, functional overlap among ADAMTS family members (ADAMTS4, ADAMTS5, ADAMTS8) provides additional redundancy that buffers against individual pathway perturbations.^[^ ^41^, ^43^, ^44^ ^]^ These observations support our central hypothesis that mechanotransduction, rather than direct vascular modulation, represents ADAMTS1's primary pathological mechanism in post‐MI remodeling. Alternative explanations for our findings should be considered. The discrepancy between in vitro endothelial dysfunction and limited in vivo vascular changes could reflect not only compensatory mechanisms, but also the possibility that our in vitro conditions may not fully recapitulate the complex in vivo environment. Additionally, while we demonstrate correlation between ADAMTS1 expression and ITGα8 activation, other parallel pathways may contribute to the observed phenotypes. The substrate‐dependent activity pattern creates a pathological specificity switch wherein ADAMTS1 remains largely inactive in healthy tissue but becomes highly active in the PG‐rich, mechanically altered post‐MI environment.^[^ ^45^, ^46^, ^47^, ^48^ ^]^ This mechanistic understanding repositions our focus toward ADAMTS1's primary mode of action: ECM‐mediated mechanotransduction that enables ECs to influence cardiac fibroblast behavior across tissue distances without requiring direct cell contact or traditional paracrine factors.

Our study identifies a mechanotransduction pathway wherein ADAMTS1‐mediated PG cleavage alters extracellular matrix mechanical properties to specifically activate cardiac fibroblast ITGα8 mechanosensing. Central to this mechanism is ITGα8's role as a specialized high‐threshold mechanoreceptor with distinct activation requirements—our hydrogel experiments demonstrate that ITGα8 exhibits preferential activation on substrates with specific elastic moduli, precisely matching the mechanical environment created by ADAMTS1‐mediated ECM remodeling.^[^ ^49^ ^]^ This contrasts markedly with other integrin family members: ITGαv responds to low‐stiffness environments and mediates tumor invasion, while ITGα5 demonstrates broader stiffness responsiveness associated with general cell migration.^[^ ^50^ ^]^

Critically, our co‐immunoprecipitation experiments indicated that ADAMTS1 and ITGα8 do not interact through direct protein binding, confirming mechanotransduction as the primary communication mode and distinguishing this pathway from conventional receptor‐ligand models.^[^ ^47^ ^]^ Instead, specificity arises from the unique combination of ADAMTS1's substrate selectivity—cleaving versican at specific sites to generate bioactive fragments and alter matrix compliance—and ITGα8's mechanical threshold requirements, creating a pathway that remains dormant in healthy tissue but becomes highly active in the PG‐rich, mechanically perturbed post‐MI environment. Our data demonstrate that ITGα8 activation leads to increased cardiac fibroblast proliferation and activation, as evidenced by increased BrdU^+^ and α‐SMA^+^ cell numbers. The specific downstream signaling pathways mediating these effects require further investigation. Upon activation, ITGα8 likely initiates downstream signaling through FAK‐Src‐PI3K/AKT pathways, promoting fibroblast survival and proliferation. Simultaneously, RhoA activation enhances contractile force generation.^[^ ^25^, ^51^ ^]^ This mechanotransduction axis integrates with biochemical pathways to enhance TGF‐β signaling through YAP/TAZ‐mediated transcription.^[^ ^52^ ^]^ The result is a feed‐forward loop that intensifies over time, differing from the transient effects of classical paracrine signaling.^[^ ^53^, ^54^ ^]^

ADAMTS1's impact on cardiac function emerges through its coordinated effects on scar size, mechanical properties, and compositional organization—three interconnected determinants that collectively govern post‐MI outcomes. Rather than viewing these as independent variables, our findings reveal how ADAMTS1 orchestrates their pathological integration through a unified mechanism centered on PG‐collagen network disruption. ADAMTS1 overexpression produced profound cardiac dysfunction through enlarged, mechanically inferior scars, while ADAMTS1 deficiency yielded protective effects with smaller, mechanically superior scars. This enlarged scar burden directly correlates with clinical outcomes, as demonstrated by MRI studies showing that extensive scarring independently predicts mortality, heart failure progression, and arrhythmic risk.^[^ ^55^, ^56^, ^57^ ^]^ Advanced imaging techniques reveal how transmural scar extent correlates with sudden cardiac death^[^ ^57^, ^58^, ^59^, ^60^ ^]^, while our model demonstrates that ADAMTS1‐mediated expansion replaces viable myocardium and disrupts electrical conduction pathways.^[^ ^61^ ^]^ Simultaneously, ADAMTS1 disrupts the natural temporal progression of scar mechanical maturation. Normal healing involves initial matrix softening followed by progressive stiffening through collagen cross‐linking and organization.^[^ ^62^, ^63^, ^64^ ^]^ ADAMTS1 overexpression interrupts this sequence, maintaining persistently reduced elastic modulus that shifts ventricular pressure‐volume relationships and elevates filling pressures, predisposing to diastolic dysfunction.^[^ ^65^, ^66^ ^]^ Clinical studies confirm this relationship, showing that reduced scar stiffness correlates with elevated filling pressures and increased heart failure hospitalization risk.^[^ ^67^ ^]^ Conversely, our ADAMTS1 knockout model produced “small but stiff” scars with preserved or enhanced modulus, optimizing ventricular performance by maintaining structural integrity while minimizing excessive wall stress.^[^ ^55^, ^63^ ^]^

The compositional dimension reveals how ADAMTS1's uneven PG degradation creates pathological heterogeneity within scar tissue. Excessive collagen deposition combined with PG fragmentation generates disorganized fiber bundles that impair cardiomyocyte alignment and compromise force transmission.^[^ ^68^, ^69^, ^70^ ^]^ These changes create osmotic imbalances that separate myocytes and disrupt gap junction function, while the heterogeneous mixing of viable myocardium with fibrotic tissue forms border zone channels that promote reentrant arrhythmias through slow conduction zones.^[^ ^71^, ^72^ ^]^ ADAMTS1 drives this heterogeneity through preferential versican cleavage that generates bioactive fragments promoting patchy collagen deposition.^[^ ^73^, ^74^, ^75^ ^]^

Our findings illuminate an apparent paradox in cardiac fibrosis: how ADAMTS1's proteolytic activity against versican ultimately leads to net ECM accumulation rather than matrix depletion. This counterintuitive outcome reflects the enzyme's generation of bioactive cleavage products that function as potent pro‐fibrotic signals. ADAMTS1‐derived versican fragments activate TLR2 receptors on fibroblasts and macrophages, initiating cascades that culminate in TGF‐β release and sustained matrix synthesis.^[^ ^76^ ^]^ This creates self‐amplifying feedback loops where proteolytic degradation paradoxically drives increased ECM production rates that exceed breakdown, resulting in progressive scar expansion.^[^ ^34^, ^40^, ^41^ ^]^

These findings may have clinical relevance for human heart failure, where ADAMTS1 upregulation in failing myocardial tissues correlates with enhanced versican cleavage and fibrotic expansion.^[^ ^34^, ^40^, ^41^ ^]^ Whether this association contributes to adverse outcomes requires further investigation, though preliminary evidence suggests potential links to ventricular arrhythmias in both ischemic and non‐ischemic heart failure subtypes.^[^ ^43^, ^56^ ^]^ Therapeutically, the substrate specificity of ADAMTS1 for pathological environments suggests it might offer more selective targeting compared to broad‐spectrum approaches.^[^ ^38^, ^77^, ^78^ ^]^

While our study elucidates a novel ADAMTS1‐ITGα8 mechanotransduction axis in post‐MI remodeling, several limitations warrant consideration. Small sample sizes may have limited detection of subtle phenotypic variations, while incomplete Cre recombination efficiency and potential compensatory pathways in genetic models could confound interpretations. The reliance on 2D hydrogels simplifies complex 3D cardiac environments, potentially overlooking spatial mechanical cues. The exclusive use of male mice restricts generalizability, given documented sex differences in cardiac remodeling where estrogen modulates integrin signaling.^[^ ^79^ ^]^ Additionally, the specificity of our Cre drivers requires further validation, as off‐target recombination could influence outcomes.

Future investigations should employ single‐cell approaches to dissect intercellular networks, incorporate female models, and explore hormonal influences on this pathway. Despite these constraints, this pathway's novelty offers a foundation for interventions that may mitigate heart failure while preserving essential repair processes.

In conclusion, our research identifies a mechanotransduction pathway between EC‐derived ADAMTS1 and cardiac fibroblast ITGα8 that contributes to post‐MI scar formation. Our data suggest that matrix remodeling enzymes can regulate cellular responses through mechanosensitive signaling. These findings indicate that the ADAMTS1‐ITGα8 axis may represent a therapeutic target for modulating cardiac remodeling, though clinical translation will require careful validation of these mechanisms in human disease.

## 4 Experimental Section

### Human Samples

Human heart samples were obtained from two sources: patients with ischemic heart failure undergoing heart transplantation and normal heart donors deemed unsuitable for transplantation due to non‐cardiac reasons. All participants provided signed, written informed consent for the use of their samples in this study. Detailed patient information relevant to this investigation is available in Table S1 (Supporting Information). This study was conducted in full compliance with the principles of the Declaration of Helsinki, and all protocols involving human heart tissue were approved by the Review Committee of the Renmin Hospital of Wuhan University (WDRY2018‐K021).

### Animal Studies

All animal experiments were conducted in accordance with the National Institutes of Health's Care and Use of Laboratory Animals guidelines and permitted by the Animal Care and Use Committee of Wuhan University Renmin Hospital (Approval No. WDRM20220704A). Only male mice were used to account for sex‐related variables and reduce experimental variability during this initial mechanistic characterization phase, although this work acknowledges that this represents a limitation affecting generalizability to female participants. Mice were housed in an SPF‐grade facility under controlled environmental conditions and were handled in accordance with blinding and randomization principles.

### Experimental Group Design

This study employed various control strategies for different experimental approaches: (1) For viral overexpression experiments: VEC^Tie^ served as the control, with both groups receiving identical viral injection procedures; (2) For conditional knockout experiments: Littermate ADAMTS1^fl/fl^ mice without Tek‐iCreER served as controls for ADAMTS1^CKO^ experiments, whereas ITGα8^fl/fl^ littermates without Col1a2‐CreER served as controls for ITGα8^CKO^ experiments; (3) For rescue experiments: ADAMTS1^Tie^ + ITGα8^fl/fl^ mice were compared to ADAMTS1^Tie^ + ITGα8^CKO^ groups.

### Genetic Mouse Models and Validation

To generate Adamts1‐overexpressing mice, wild‐type mice received orbital vein injections of adeno‐associated virus 9 (AAV9) carrying endothelial‐specific Adamts1 (ADAMTS1^Tie^) or control vector (VEC^Tie^) 1 week pre‐surgery (5 × 10^11^ pfu/mouse). EC‐specific Adamts1 knockout mice were created by crossing Adamts1^fl/fl^ mice (Cyagen Biosciences, #S‐CKO‐01052/CKOAIP230210JF1) with Tek‐iCre mice (Gempharmatech, T003764). Additionally, CF‐specific ITGα8 knockout mice were generated by breeding ITGα8^fl/fl^ mice (Gempharmatech, T024282) with Col1a2^CreER^ mice (Jackson Laboratory, #02 9567). The Col1a2‐CreER system was selected for its validated specificity in targeting collagen‐producing fibroblasts with temporal control through tamoxifen induction.^[^ ^80^, ^81^ ^]^

ADAMTS1^fl/fl^ and ITGα8^fl/fl^ mice were obtained by cross‐breeding the corresponding heterozygous mice (ADAMTS1^fl/‐^ and ITGα8^fl/‐^). To ensure that genotypes required for the experiment, these were identified by PCR with specific primers. The primer sequences targeting the ADAMTS1 mutant allele were designed based on the 5′ arm loxp site (F1, 5′‐TGATCTCATTTCTGTGGCCTTCC‐3′ and R1, 5′‐ATCAGTCCAGGCTAACATTCCTCA‐3′) and the 3′ arm loxp site (F2, 5′‐ATCTGTATTGCCTCATTTCCTTTCG‐3′ and R2, 5′‐GACTTGAAGGACTGACTCTAAAGC‐3′). Upon identification of homozygous mice, a 284 bp‐ and a 218 bp‐ positive fragment can be obtained. Successful generation of ADAMTS1^CKO^ mice need to be validated by both primers targeting ADAMTS1 (mentioned above) and Tek‐iCre (forward primer, 5′‐GGGCAGTCTGGTACTTCCAAGCT‐3′ and reverse primer, 5′‐CTTGATTCACCAGATGCTGAGGTTA‐3′, targeted band can be observed at 411 bp position). Similarly, the primer sequences targeting the ITGα8 on 5′ and 3′ arm loxp sites are 5′ arm‐F1‐5′‐TTATCCTGGGATCCTGCTGTCCTA ‐3′; 5′ arm‐R1‐5′‐TGGTGAAAGAAAGTGGACCAGGT‐3′ with target band at 423bp and 3′ arm‐F1‐5′‐TCTGAGGCGGAAAGAACCAG‐3′; 3′ arm‐R1‐5′‐TTTCCACTTGTATGCCACACCAC‐3′ with target band at 260 bp. ITGα8^CKO^ mice are validated by primers targeting ITGα8 and Col1α2 (forward primer, 5′‐CATGTCCATCAGGTTCTTGC‐3′ and reverse primer, 5′‐TGAAAAAGTCCACTAATTAAAACCA‐3′ with a targeted band at about 200 bp).

The Col1a2‐CreER system was selected based on extensive literature validation. Kanisicak et al. demonstrated that Col1a2‐CreER effectively targets CFs with minimal off‐target effects in cardiomyocytes or ECs. Park et al. further validated this system for studying fibroblast activation in cardiac fibrosis models. The Col1a2 promoter drives expression specifically in collagen‐producing cells, making it particularly suitable for targeting activated fibroblasts during cardiac remodeling. While alternative drivers such as Postn‐Cre or Tcf21‐Cre exist, Col1a2‐CreER provides superior temporal control through tamoxifen induction.

Knockout efficiency was validated through multiple approaches. For ADAMTS1 conditional knockout, IF analysis using cell‐type‐specific markers showed approximately substantial reduction in ADAMTS1 protein expression specifically in CD31⁺ ECs. For ITGα8 conditional knockout, IF assessment in α‐SMA⁺ CFs showed similar knockout efficiency. These efficiency levels were consistent with expected tamoxifen‐inducible Cre recombination performance, which typically achieves 70–85% efficiency under optimal conditions.

EC specificity of AAV9‐Tie2 system was assessed through multiple approaches. Cell‐type specific expression analysis was performed using qRT‐PCR on isolated cardiac cell populations, demonstrating significant ADAMTS1 upregulation in CD31⁺ ECs with minimal changes in CFs or cardiomyocytes. IF co‐localization studies showed >80% of ADAMTS1⁺ cells co‐expressing CD31 in experimental conditions. While this work acknowledges that the Tie2‐Cre system may have some activity in certain hematopoietic lineages, the consistency of phenotypic observations with endothelial‐derived effects and the indirect nature of the proposed mechanism support endothelial‐specific targeting as the primary driver of observed effects.

### Myocardial Infarction Model and Interventions

For the establishment of the MI model via left anterior descending coronary artery ligation, mice were initially anesthetized with pentobarbital sodium (80 mg kg^−1^, Sigma‐Aldrich, P3761, USA) administered via intraperitoneal injection, with sham operations serving as controls. Subsequently, tamoxifen (50 mg kg^−1^) was administered to the treatment groups 1 week prior to and following the surgery to induce *ADAMTS1* and *ITGα8* deletion in ECs and CFs, respectively. This dosage was selected based on validated protocols achieving >85% recombination efficiency, while avoiding cardiotoxic effects associated with higher doses.^[^ ^82^ ^]^

### Cardiac Function Assessment

For cardiac function assessment, echocardiography was performed using a FUJIFILM Visualsonics Vevo3100 system with a 21–44 MHz probe (axial resolution 50 µm) prior to tissue collection. Mice were anesthetized with 4% isoflurane in 0.5 L min^−1^ oxygen. Thereafter, electrocardiogram and respiratory data were monitored via a conductive adhesive‐coated physiological monitoring table. M‐mode images in the parasternal short‐axis view were acquired to measure the left ventricular internal diameters during diastole (LVIDd) and systole (LVIDs). Subsequently, the endocardial (Endo) trajectories were traced to calculate the LV EDV, ESV, stroke volume, EF, and FS. Additionally, B‐mode speckle tracking analysis of Endo and epicardial (Epi) motion in long‐axis views provided the global longitudinal strain (GLS) and regional metrics. The latter included the longitudinal and radial strain rates (1/s), displacement (mm), and strain (%) of Epi/Endo in six segments—posterior as well as anterior base, mid, and apex.

### Tissue Collection

At the experimental endpoints, the mice were euthanized by cervical dislocation under deep anesthesia induced by 2.5% isoflurane inhalation. Heart tissues were either fixed in 4% paraformaldehyde or frozen in liquid nitrogen for subsequent pathological and molecular analyses.

### Cell Isolation and Culture

This study utilized various sources of ECs and fibroblasts for in vitro experiments. CFs from ITGα8^fl/fl^ and ITGα8^‐/‐^ mice were isolated using a Langendorff‐free method adapted from previous studies.^[^ ^83^ ^]^ Briefly, genetically identified mouse hearts were enzymatically digested with Collagenase II/IV (0.5 mg mL^−1^, Worthington) and Protease XIV (0.05 mg mL^−1^, Sigma‐Aldrich). CFs were isolated through differential adherence. Cardiac ECs were extracted from wild‐type mice hearts after removing connected vascular tissues. Hearts were digested in Ca^2+^ and Mg^2+^‐free HBSS (Gibco) supplemented with antibiotics. Cell suspensions were incubated with KRT8/803 antibody (Abcam) to block Fc receptors, followed by APC Rat Anti‐Mouse CD31 antibody (BD Pharmingen) conjugated to magnetic beads (Miltenyi Biotec). ECs were isolated using a MACS Dissociator (Miltenyi Biotec). Both CFs and ECs were cultured on laminin‐coated (5 µg mL^−1^, Roche, Switzerland) dishes. HUVECs (YRGene) were cultured in RPMI 1640 medium with 10% FBS. Mouse ECs were maintained in Krebs–Hensleit's Solution with 15% FBS and 1% penicillin‐streptomycin. NRCFs and CFs were cultured in DMEM/F12 (1:1) supplemented with 15% FBS.

### In Vitro Experiments

To assess ADAMTS1 expression in ECs and CFs, NRCFs, HUVECs, and mouse ECs were utilized. HUVECs and NRCFs were exposed to normoxic (5% O_2_) or hypoxic (1% O_2_) conditions for 24 h using a Tri‐gas Incubator (Healforce, China), followed by ADAMTS1 mRNA quantification. IF staining was performed on ECs and NRCFs under similar conditions to evaluate ADAMTS1 localization and expression. To investigate ADAMTS1‐mediated ITGα8 activation, two experiments were conducted: (1) NRCFs were treated with recombinant human ADAMTS1 (100 ng mL^−1^, R&D Systems) or PBS control, followed by co‐immunoprecipitation and IF staining to assess ADAMTS1‐ITGα8 interaction and activation. (2) NRCFs were cultured on 1%, 2%, or 3% hydrogels for 24 h to evaluate ITGα8 activation in response to altered substrate stiffness using IF staining. Additionally, this work used ITGα8^fl/fl^ mice and AAV9‐Cre‐mediated in vitro knockout to assess the impact of ITGα8 deficiency on CF contractility. Detailed protocols for these experiments are provided in the respective Experimental Section.

### Tube Formation Assay

Cardiac ECs isolated from VEC^Tie^ and ADAMTS1^Tie^ mice were seeded on Matrigel‐coated 96‐well plates (Corning, #356 234) at 8 × 10⁴ cells per well in serum‐free DMEM. After 6 h incubation at 37 °C, tube formation was assessed by phase‐contrast microscopy. Images were captured using a Leica microscope and analyzed using ImageJ software with the Angiogenesis Analyzer plugin. Total tube length and the number of junctions were quantified across five random fields per well.

### Wound Healing Migration Assay

Confluent EC monolayers in 24‐well plates were scratch‐wounded using a sterile 200 µL pipette tip. Cells were washed with PBS to remove debris and cultured in serum‐free medium. Images were captured at 0, 12, and 24 h using phase‐contrast microscopy. Migration rate was calculated as: (initial wound area − final wound area)/initial wound area × 100%.

### Cell Viability Assay

Cell viability was assessed using the MTT assay (Sigma‐Aldrich, #M5655) at 24, 48, and 72 h according to the manufacturer's instructions. Absorbance was measured at 570 nm using a microplate reader. Results are expressed as percentage of control values.

### Co‐Immunoprecipitation (Co‐IP) of Adamts1 and ITGα8

NRCFs were lysed in 100 µL of lysis buffer and sonicated. Concurrently, Adamts1 antibody was incubated with protein A/G magnetic beads (HY‐K0202, MCE). The cell lysate supernatant was divided for Input and IP samples. The antibody‐bead complex was added to the IP samples and incubated at room temperature for antigen adsorption. Beads‐antibody‐antigen complexes were magnetically separated (HY‐K0200, MCE) and eluted with 0.15 m Glycine, 0.5% Triton X‐100, pH 2.5. The eluate was neutralized with 0.1 m NaOH (10% v/v). Proteins were denatured by heating and analyzed by SDS‐PAGE electrophoresis.

### Preparation of Stiffness‐Tunable Sodium Alginate Hydrogels

To validate the mechanical activation of ITGα8 by Adamts1, tunable stiffness hydrogel matrices were we prepared as previously described.^[^ ^84^, ^85^, ^86^ ^]^ Three SA concentrations (1%, 2%, and 3% w/v) were selected to create a gradient of substrate stiffness appropriate for cardiac mechanotransduction studies.

SA solutions (Aladdiin) were prepared at concentrations of 1%, 2%, and 3% (w/v) in sterile water, poured into gel‐forming molds, and cross‐linked with 0.5 m CaCl_2_ solution for 30 min at room temperature. Hydrogel mechanical properties were characterized using a MARK‐10 Force/Torque Indicator (Series‐5). For mechanical testing, cylindrical hydrogel samples were prepared with standardized dimensions (diameter: 10 mm, height: 5 mm) to ensure consistent testing conditions. Young's modulus measurements were performed by applying controlled compression forces and recording the corresponding deformation. Force–displacement data were converted to stress–strain relationships, and the Young's modulus was calculated from the linear slope of the stress–strain curve in the 0% to 10% strain range (*n* = 6 per concentration). Only measurements showing linear behavior (*R* ^2^ ≥ 0.90) within this strain range were included in the analysis.

Testing was conducted at room temperature with samples maintained in a hydrated state to prevent dehydration artifacts. Each sample was subjected to incremental loading with force measurements recorded at defined displacement intervals. The elastic modulus was determined using the formula: *E* = (Δ*σ*/Δ*ε*) × 100, where Δ*σ* represents the change in stress and Δ*ε* represents the change in strain within the 0–10% strain range.

For cell culture experiments, hydrogels were prepared using identical cross‐linking conditions and cut to fit 24‐well plates. Hydrogels were washed with sterile water and UV‐sterilized for 2 h. After PBS washes and equilibration with DMEM/F12 1:1, hydrogels were coated with rat tail tendon type 1 collagen (0.012 mg mL^−1^, Solarbio C8062 in 0.36 g L^−1^ acetic acid) for 1 h to promote cell adhesion. NRCFs were seeded onto the prepared hydrogels at a density of 2 × 10⁴ cells cm^−^^2^. ITGα8 activation and cellular responses to varying mechanical forces were assessed by in situ immunofluorescence (ISI) staining after 24 and 48 h of culture.

### Contraction Force Detection of Myofibroblasts by Silicon Nanopillar Array

Si‐NPs were provided by the Beijing Institute of Nanoenergy and Nanosystems (Chinese Academy of Sciences, Beijing). Mechanical and physical properties were characterized using SEM (SU8020, Hitachi, Japan). The average nanowire length was 2926 ± 55 nm with a diameter of 304 ± 4 nm. COMSOL finite element simulations yielded a linear force‐displacement relationship of Force = 0.00639 (µN nm^−1^) × displacement (nm) for forces between 0–10 µN. Detailed Si‐NP properties have been previously reported.^[^ ^87^, ^88^ ^]^

Si‐NPs were prepared by overnight soaking in 5% hydrochloric acid, followed by five rinses with purified water. They were then placed in 24‐well cell culture plates, immersed in 75% ethanol for 2 h, and UV‐sterilized (400 MW m^−2^, HFsafe‐900, China). After rinsing with sterile deionized water, Si‐NPs were equilibrated in serum‐free DMEM/F12 1:1 (12 634 010, ThermoFisher Scientific, USA) for 1 h before use.

Myofibroblasts isolated from ITGα8^fl/fl^ and ITGα8^‐/‐^ mice (referred to the method mentioned above) were individually seeded into 24‐well cell culture plates (approximately 5 × 10^3^ cells every well). After 24 h incubation with DEMEM/F12 1:1 containing 15% fetal bovine serum (FBS, 26 010 074 from ThermoFisher Scientific), culture media was replaced with 1/2 volume of serum‐free medium. Meanwhlie, myofibroblasts were administrated with AAV9‐Cre (50 MOI, 5 × 10^10^ pfu mL^−1^, BB040 from GENERAL BIOL, CHN) for 4 h. And then, replace with 15% FBS DEMEM/F12 1:1 for another 20 h. Eventually, cell contractility was assayed against Si‐NP after stimulating the myofibroblasts with 1% hydrogel for 24 h.

Samples were fixed with glutaraldehyde for 2 h at room temperature. Si‐NPs were then dehydrated through a graded ethanol series (50% to 100%, 15 min each step). After gold‐coating by vacuum sputtering, the morphology of myofibroblasts attached to Si‐NPs was examined using SEM. All images were analyzed using ImageJ software.

### Molecular and Biochemical Assays—Quantitative Real‐Time PCR

Total RNA was isolated from heart samples and cells as described above or in our previous studies.^[^ ^89^ ^]^ cDNA was synthesized using reverse transcription applications (#4 374 967, ThermoFisher, USA). Quantitative real‐time PCR was performed using a Light Cycler 480 (Roche) following our established protocol.^[^ ^90^ ^]^ Relative gene expression was calculated using the 2^‐∆∆Ct^ method, with GAPDH as the internal reference. Primer sequences for all target genes are listed in Table S5 (Supporting Information).

### Protein Extraction and Western Blot

Total protein was extracted using RIPA lysis buffer (P0013B, Beyotime, China) supplemented with protease inhibitors (cOmplete, PhosSTOP, and PMSF; Merck, USA). Membrane proteins were isolated using a Membrane and Cytosol Protein Extraction Kit (P0033, Beyotime) according to the manufacturer's instructions, with additional sonication (5 Hz for 10 s, XFSTPRP‐CL‐24, Misonix, Germany) to enhance protein yield. Protein concentrations were determined using Pierce BCA Protein Assay kits (A55864, ThermoFisher Scientific, USA). Equimolar samples were prepared with loading buffer and denatured at 70 °C before SDS‐PAGE. Proteins were transferred to polyvinylidene difluoride (PVDF) membranes using a Trans‐Blot Semi‐Dry Electrophoretic Transfer Cell (10 V, 2.5 A, 10 min; 1 703 940, Bio‐Rad, USA). Membranes were blocked with 3% skim milk or bovine albumin buffer and incubated overnight at 4 °C with primary antibodies (listed in Table S6, Supporting Information). After washing, membranes were incubated with species‐appropriate secondary antibodies (1:10 000, SA00001‐1‐A, and SA00001‐2, Proteintech) for 1 h at room temperature. Protein bands were visualized using enhanced chemiluminescence kits (E411‐04, Vazyme) and imaged with a ChemiDoc MP Imaging System (Bio‐Rad). Image quantification and adjustment were performed using Image Lab 6.0 software.

### Histological and Imaging Analyses—In Situ Immunofluorescence (ISI) Staining

Heart samples collected 2 weeks post‐MI were fixed, paraffin‐embedded, and sectioned (5 µm thickness). After dewaxing and hydration, antigen retrieval was performed using citrate buffer. Sections were blocked with 10% goat serum (16 210 064, Gibco) to prevent non‐specific binding. Primary antibodies were incubated for 2 h at 37 °C. Adamts1 antibody was used to detect its location and expression level. CD31 antibody labeled ECs. Collagen deposition was assessed using collagen 1a1, collagen 3a1, and vimentin antibodies. Myofibroblast activation was detected using Postn and α‐SMA antibodies, while proliferation was assessed with 5‐bromodeoxyuridine (BrdU). PGs aggregation was indicated by Decorin and VCAN staining. Versikine, a VCAN fragment degraded by Adamts1, was detected to indirectly confirm ADAMTS1^Tie^ efficiency. (Detailed antibody information is provided in Table S6, Supporting Information). Species‐appropriate secondary antibodies (Alexa Fluor 568 and 488, Invitrogen) were applied, followed by DAPI (C1005, Beyotime) for nuclear staining. Images were acquired using Leica confocal microscopes (STELLARIS 5 and STELLARIS 8, Germany) and processed with Photoshop 2021 and LAS X software.

### Angiogenesis Assessment—Capillary Density Analysis

Capillary density analysis was performed in transverse sections of the periinfarct zone from left ventricles 14 days after MI. Hearts were collected, fixed in 4% paraformaldehyde, and processed for paraffin embedding as described above. Serial sections (5 µm thickness) were prepared and subjected to fluorescent immunohistochemical staining.

Capillary density was evaluated using platelet and EC adhesion molecule‐1 (CD31; endothelial marker) IF staining. The periinfarct zone was defined as the border region within 1 mm of the infarct boundary. For each animal, five random high‐power fields (×400 magnification) were analyzed in the periinfarct zone. The number of capillaries per square millimeter was counted in a blinded fashion using ImageJ software. Only structures with clearly identifiable lumens and appropriate endothelial morphology were included in the analysis. Results are expressed as capillary counts per mm^2^ of tissue area.

### ECM Scanning by Transmission Electron Microscope (TEM)

Cardiac tissue samples collected 2 weeks post‐MI were fixed in 2.5% glutaraldehyde (EM Grade, Solarbio) overnight at 4 °C. 1% OsO_4_ fix solution was added for 90 min and then dip the samples in 2% uranyl acetate solution for 2 h at low temperature. After dehydrating in a graded ethanol solution, the samples were embedded in epoxy resin (Merck). Ultrathin sections (approximately 50 nm) were prepared using a Leica EM UC7 ultramicrotome (Germany) and imaged with a Hitachi HC‐1 transmission electron microscope at 80 kV (Japan). Images were processed using ImageJ and Adobe Photoshop 2021.

### Young's Modulus Detection by Atomic Force Microscope

Cardiac tissue samples collected 2 weeks post‐MI were snap‐frozen in liquid nitrogen and serially sectioned (20 µm thickness) using a NX50 cryostat (ThermoFisher Scientific).^[^ ^55^ ^]^ To facilitate IA identification, one section was immunostained for Col1a1 and Col3a1. AFM measurements were performed using a Bruker Dimension Icon system equipped with Tespa‐v2 cantilevers (resonance frequency = 320 kHz, force constant = 42 N m^−1^, tip radius = 8 nm). Force–displacement curves were acquired using Hertzian‐Spherical and Sneddon‐Conical models.^[^ ^91^, ^92^ ^]^ Measurement parameters were: contraction speed = 1.0 µm s^−1^, applied force = 20 nN, and contraction distance = 500 nm. At least 50 sites per sample were analyzed. Raw AFM data were processed using Gwyddion software, while statistical analysis and visualization were performed using GraphPad Prism 9.4.1.

### Omics Analyses—RNA‐Sequencing Assay

Heart tissues from the IA of mice 2 weeks post‐MI and sham‐operated controls (*n* = 3–5) were collected. Total RNA was extracted using TRiozl (Invitrogen Life Technologies, USA), Trichloromethane (Sigma‐Aldrich) and isopropanol (ThermoFisher, USA). RNA quality and quantity were assessed using a Nanodrop ND2000 spectrophotometer, Qubit 4.0, and Agilent 2100/4200 system. Sequencing and differential expressed genes (DEGs) analysis were performed by Bioyi Biotechnology Co., Ltd. (Wuhan, China). Libraries were constructed and sequenced on the DNBSEQ‐T7 platform with PE150 mode. Raw reads were filtered using fastp (v0.21.0), mapped to the genome with HISAT2 (v2.1.0), and quantified using StringTie (v2.1.5). DEGs analysis was performed using DESeq2 (v1.30.1), with significance criteria of |log_2_FoldChange| > 1 and padj ≤ 0.05.

For comparative analysis, human sample data (GSE132143) were retrieved from the GEO database.^[^ ^93^ ^]^ DEGs were identified using “DESeq2” and “dplyr” packages in R. Joint analysis was performed using Metascape (https://metascape.org/gp/), with the top ten enrichment pathways selected based on “_LogP_GSE132143” and “_LogP_Personal” significance.^[^ ^94^ ^]^ For ADAMTS1 overexpressing mice, GO and KEGG enrichment analyses were conducted using R and Metascape, with scar formation‐related pathways and genes annotated. Bioinformatics data visualization was performed using R 4.3.1 and GraphPad Prism 9.4.1.

### Proteomic Profiles of Scar Tissue

Heart tissues from ADAMTS1^CKO^ and ADAMTS1^fl/fl^ mice were collected 2 weeks post‐MI and pulverized in liquid nitrogen. The resulting powder was lysed in a fourfold volume of buffer containing 8 m urea (Sigma‐Aldrich) and 1% protease inhibitor cocktail (Merck). After sonication, protein concentration was determined. Equal amounts of protein from each sample underwent trypsin digestion. Proteins were precipitated with 20% trichloroacetic acid (TCA, Sigma‐Aldrich) at 4 °C for 2 h, washed thrice with pre‐cooled acetone (HANNOL, China), and lyophilized. The protein pellets were resuspended in 200 × 10^−3^ m tetraethylammonium bromide (TEAB, Sigma‐Aldrich) and digested overnight with 2% (w/w) trypsin (Promega, China).

Peptide solutions were reduced with 5 × 10^−3^ m DL‐dithiothreitol (DTT, Sigma‐Aldrich) for 30 min at 56 °C, followed by alkylation with 11 × 10^−3^ m iodoacetamide (Sigma‐Aldrich) for 15 min at room temperature in the dark. The resulting peptides were labeled with TMT reagent (ThermoFisher Scientific) for 2 h according to the manufacturer's instructions. Samples were desalted using a C18 column (Strata‐X, Phenomenex, USA), vacuum‐dried, and reconstituted in phase A (0.1% formic acid, 2% acetonitrile in water). High pH reversed‐phase HPLC fractionation was performed using a ZORBAX 300Extend C18 column (5 µm particles, 4.6 mm ID, 25 cm length; Agilent, USA) with a gradient of 8–32% acetonitrile (pH 9.0) over 60 min. The TMT‐labeled peptides were separated into 60 fractions, which were then consolidated into six fractions. These fractions were lyophilized and subsequently analyzed by LC‐MS/MS.

Peptides were dissolved in phase A (0.1% formic acid, 2% acetonitrile in water) and separated using an EASY‐nLC 1200 Ultra HPLC system (ThermoFisher Scientific). A gradient elution was performed with phase B (0.1% formic acid, 90% acetonitrile in water) as follows: 0–4 min, 7–10% B; 4–53 min, 10–32% B; 53–57 min, 32–80% B; 57–60 min, 80% B, at a flow rate of 500 nL min^−1^. Peptide analysis was conducted on an Orbitrap Exploris 480 mass spectrometer (ThermoFisher Scientific). The ion source voltage was set to 2.3 kV with a FAIMS compensation voltage of −45V. MS1 scans were acquired at 400–1200 m/z with a resolution of 60 000, while MS2 scans started at 110 m/z with a resolution of 15 000. The automatic gain control (AGC) target was set to 100% (signal threshold 5E4 ions s^−1^, maximum injection time set to Auto). Data‐dependent acquisition (DDA) mode was used with a dynamic exclusion time of 30 s for tandem MS scans to avoid repeated parent ion scanning.

LC‐MS/MS raw data were processed using MaxQuant search engine (v.1.6.15.0) and searched against the SwissProt human database (20422 entries). Mass tolerance was set to 20 ppm for precursor ions in the first search, 5 ppm in the main search, and 0.02 Da for fragment ions. The false discovery rate (FDR) was adjusted to <1%. LC‐MS/MS analysis and bioinformatics support were provided by PTM BIO.

### Computational Analyses—Protein Docking by ZDOCK

To investigate the interaction between Adamts1 and ITGα8, we performed protein‐protein docking simulations. The 3D structures of human Adamts1(UniProt: Q9UHI8) and ITGα8 (UniProt: P53708) were obtained from UniProt (https://www.uniprot.org/) and refined using AlphaFold predictions.^[^ ^95^, ^96^ ^]^ Protein‐protein docking was conducted using the ZDOCK online server (https://zdock.umassmed.edu/).^[^ ^97^ ^]^ Binding site predictions were based on the 3D structures and physicochemical properties of both proteins. Detailed information on polar bonds and amino acid interactions was visualized and analyzed using PyMOL (Details are showed in Table S7, Supporting Information). This approach allowed us to predict potential binding sites between Adamts1 and ITGα8, providing insights into their molecular interactions.

### Statistical Analysis

All data were analyzed and visualized using GraphPad Prism 9.4.1 and OriginPro 2021b. Continuous variables are expressed as mean ± standard deviation (SD) and presented as scatter plots, line charts, histograms, or box plots with quartiles where appropriate. Comparisons between two groups were assessed using two‐tailed *t*‐tests, while one‐way analysis of variance (ANOVA) followed by Bonferroni post‐hoc tests was used for comparisons among three or more groups. Furthermore, survival curves were generated using Kaplan–Meier analysis, and group comparisons were performed using log‐rank tests. Unpaired two‐tailed Student's *t*‐test and multiple unpaired *t*‐tests (correction with two‐stage step‐up method of Benjamini, Krieger, and Yekutieli) were utilized to compare two groups. Two‐way ANOVA followed by Bonferroni's multiple comparisons test was performed in two‐factor group comparisons. Pearson correlation coefficients (two‐tailed *p*‐value and 95% confidence interval) were performed for exploring correlations between ADAMTS1 and Col1/3 or α‐SMA. Statistical significance was defined as *p* ≤ 0.05.

Data parameters are described including number of samples (*n*) and the *p*‐values are marked in the figures. Experimental images were processed using Photoshop 2021, ImageJ, Image Lab 6.0, Image Scope X64, LAS X, PyMOL 2.5.0, MESUR gauge Plus, and RStudio. All experiments were randomized, and investigators were blinded to assignment as much as possible during the experiment and outcome assessment.

## Conflict of Interest

The authors declare no conflict of interest.

## Author Contributions

C.Y.K., Z.G., and Y.L.M. contributed equally to this work. Q.Z.T., C.Y.K., Z.G., and Y.L.M. supervised and conceptualized the study. C.Y.K., Z.G., and Y.L.M. conducted experiments. C.Y.K. and Z.G. designed and performed the majority of the experiments and analyzed the majority of the data. C.Y.K., Z.G., and Y.L.M. wrote and edited the manuscript. M.Y.W., H.Y.N., P.W., W.J.Q., Z.Y., and B.S. assisted with some experiments, and discussed the results. All authors edited and approved the manuscript.

## Supporting information



Supporting Information

Supplemental Table 1

Supplemental Table 2

## Data Availability

The data supporting the findings in manuscript and associated supplementary materials are presented in Source data files providing with this paper. Any additional raw data are available from the corresponding author upon reasonable request. The datasets generated for the RNA‐seq are available through the Gene Expression Omnibus (https://www.ncbi.nlm.nih.gov/geo/query/acc.cgi?acc=GSE). The Proteomic data involved in this study can be acquired in accession code ID:
