# Supplementary material for: Adamts1 Exacerbates Post‐Myocardial Infarction Scar Formation via Mechanosensing of Integrin α8
Source: Adv Sci (Weinh). 2025 Sep 27;12(46):e04138. doi: 10.1002/advs.202504138 (PMC12697874; doi:10.1002/advs.202504138)
Supplement: Supplementary file 1 — Supporting Information [file ADVS-12-e04138-s001.docx]

# Supplementary Materials

**Adamts1 Exacerbates Post-Myocardial Infarction Scar Formation via Mechanosensing of Integrin α8**

Chun-Yan Kong, MD, PhD^1,2,3*^, Zhen Guo, MD, PhD^1,2,4,5*^, Yu-Lan Ma, MD^1,2*^, Ming-Yu Wang, MD^1,2^, Hai-Yang Ni, MD^1,2^, Pan Wang, BS^1,2^, Wen-Jun Qiu, BS^1,2^, En-Gui Wang, PhD^6^, Zhou Li, PhD^6^, Zheng Yang, MD, PhD^1,2^, Bo Shen, MD, PhD^1,2^, Qi-Zhu Tang, MD, PhD^1,2,3^**^#^**,

1 Department of Cardiology, Renmin Hospital of Wuhan University, Wuhan 430060, RP China (CY.K., Z.G., YL. M., MY.W., HY.N., P.W., WJ.Q., Z.Y., B.S., and QZ.T.).

2 Hubei Key Laboratory of Metabolic and Chronic Diseases, Wuhan, 430060, RP China (CY.K., Z.G., YL. M., MY.W., HY.N., P.W., WJ.Q., Z.Y., B.S., and QZ.T.).

3 TaiKang Center for Life and Medical Sciences, Wuhan University, Wuhan 430071, RP China (CY.K. and QZ.T.)

4 Institute of Myocardial Injury and Repair, Zhongnan Hospital of Wuhan University, Wuhan 430071, RP China (Z.G.).

5 Department of Cardiology, Zhongnan Hospital of Wuhan University, Wuhan 430071, RP China (Z.G.).

6 Beijing Key Laboratory of Micro-Nano Energy and Sensor, Beijing Institute of Nanoenergy and Nanosystems, Chinese Academy of Sciences, Beijing, 101400, China (EG.W. and Z.L.).

*These authors contributed equally to this work: CY.K., Z.G., and YL.M.

#Correspoonding Author: QZ.T.

Address for correspondence:

Qi-Zhu Tang

Department of Cardiology, Renmin Hospital of Wuhan University,

Cardiovascular Research Institute,

Hubei Key Laboratory of Metabolic and Chronic Diseases,

Wuhan University at Jiefang Road 238, Wuhan 430060, RP China.

Tel: +86 027-88073385. Email: *[qztang@whu.edu.cn](mailto:qztang@whu.edu.cn)*

#### Supplementary Figures

####
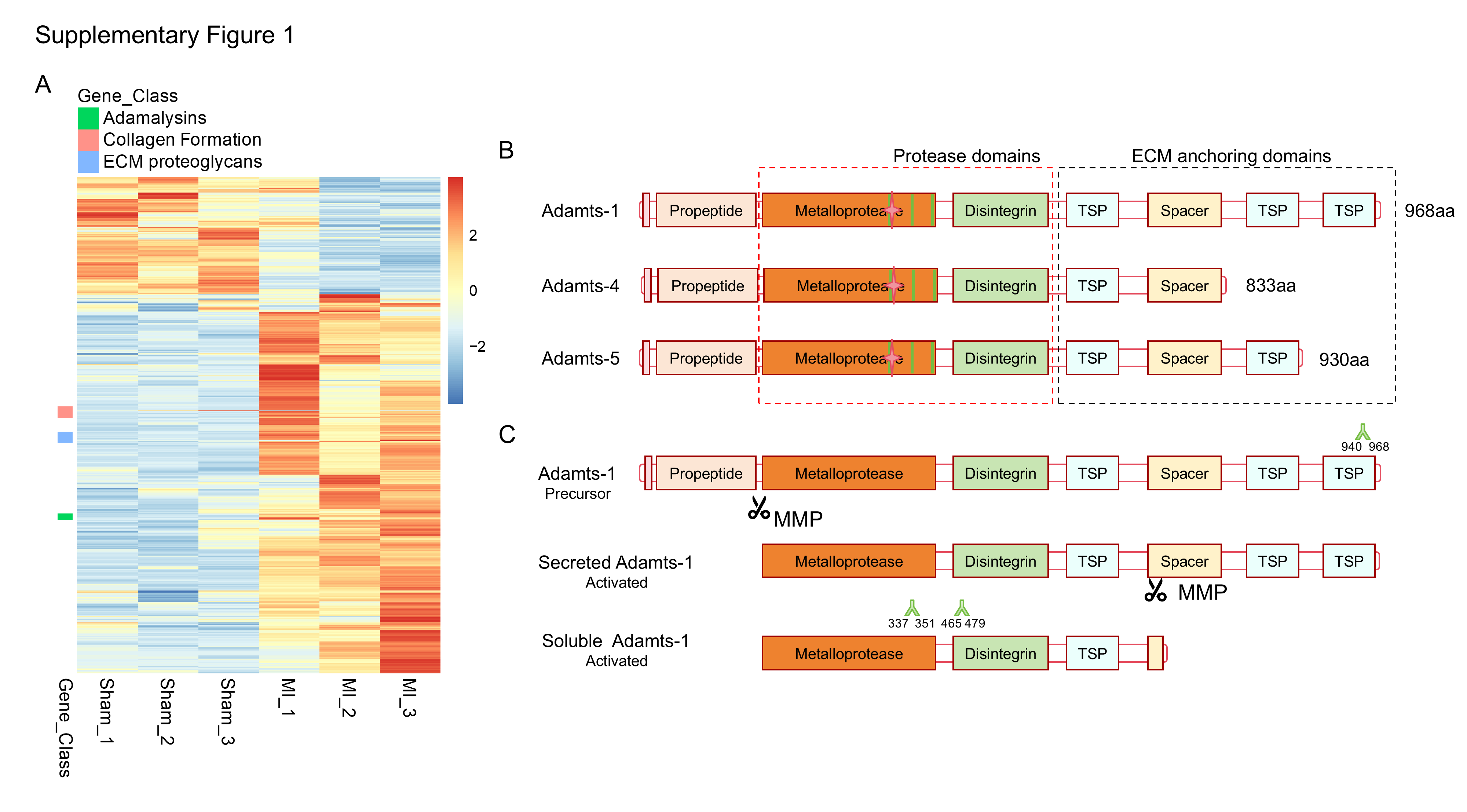
Supplementary Figure 1. Adamts1 Involvement in Post-Myocardial Infarction Scar Formation

**(A)** Heatmap depicting differential expression of Adamts-related genes following myocardial infarction (n=3 per group).

(**B)** Schematic illustration of the protein structures of Adamts1, Adamts4, and Adamts5.

(**C)** Diagrammatic representation of Adamts1 forms present in biological systems. Adamts1, a disintegrin and metalloproteinase with thrombospondin motif 1; ECM, extracellular matrix; MI, myocardial infarction; MMP, matrix metalloproteinases; TSP, thrombospondin.


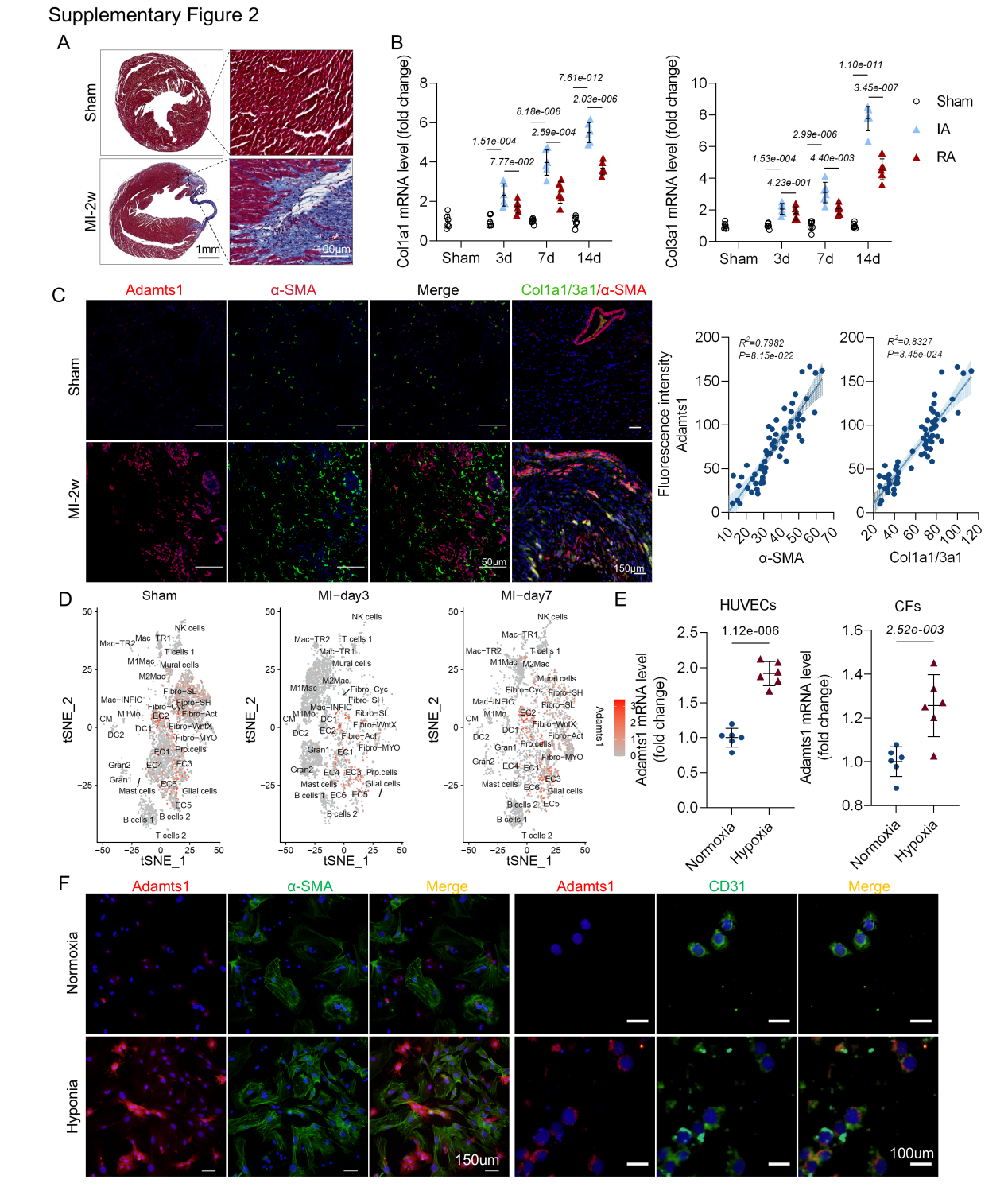


#### Supplementary Figure 2. Adamts1 Localization in Endothelial Cells Correlates with Post-MI Fibrosis Levels

**(A)** Representative Masson's trichrome staining showing microscopic changes in cardiac tissue from MI and sham-operated groups.

**(B)** qRT-PCR analysis of fibrosis markers Col1a1 and Col3a1 transcription at various time points post-MI (n=6 per group). Statistical significance: Col1a1 (3d: F(2,15)=15.77, P=2.05×10⁻⁴; 7d: F(2,15)=54.80, P=1.27×10⁻⁷; 14d: F(2,15)=208.8, P=1.12×10⁻¹¹); Col3a1 (3d: F(2,15)=16.69, P=1.53×10⁻⁴; 7d: F(2,15)=31.25, P=4.48×10⁻⁶; 14d: F(2,15)=196.1, P=1.77×10⁻¹¹).

**(C)** In situ immunofluorescence (ISI) staining and quantification showing Adamts1 expression and localization in cardiac tissue, with correlation with CF markers α-SMA, Col1a1, and Col3a1 (n=6 per group).

(**D)** Single-cell database analysis of Adamts1 cellular localization and differential expression post-MI (data from https://clara.baker.edu.au/; Farbehi N et al.,2019).

(**E)** qRT-PCR analysis of Adamts1 transcription in HUVECs and CFs under hypoxic and normoxic conditions (n=6 per group; HUVECs: t=10.39; CFs: t=3.999).

(**F)** Immunofluorescence staining showing Adamts1 levels and localization in CFs and HUVECs under hypoxic and normoxic conditions (n=6 and 3, respectively). Statistical analyses were performed using one-way ANOVA followed by Bonferroni post hoc test (**B**), Pearson's r-correlation (**C**), and Student's t-test (**E**). Data are presented as mean±SD. Adamts1, a disintegrin and metalloproteinase with thrombospondin motif 1; α-SMA, α-smooth muscle actin; CFs, cardiac fibroblasts; Col1a1, collagen type I alpha 1 chain; Col3a1, collagen type III alpha 1 chain; HUVECs, human umbilical vein endothelial cells; MI, myocardial infarction.


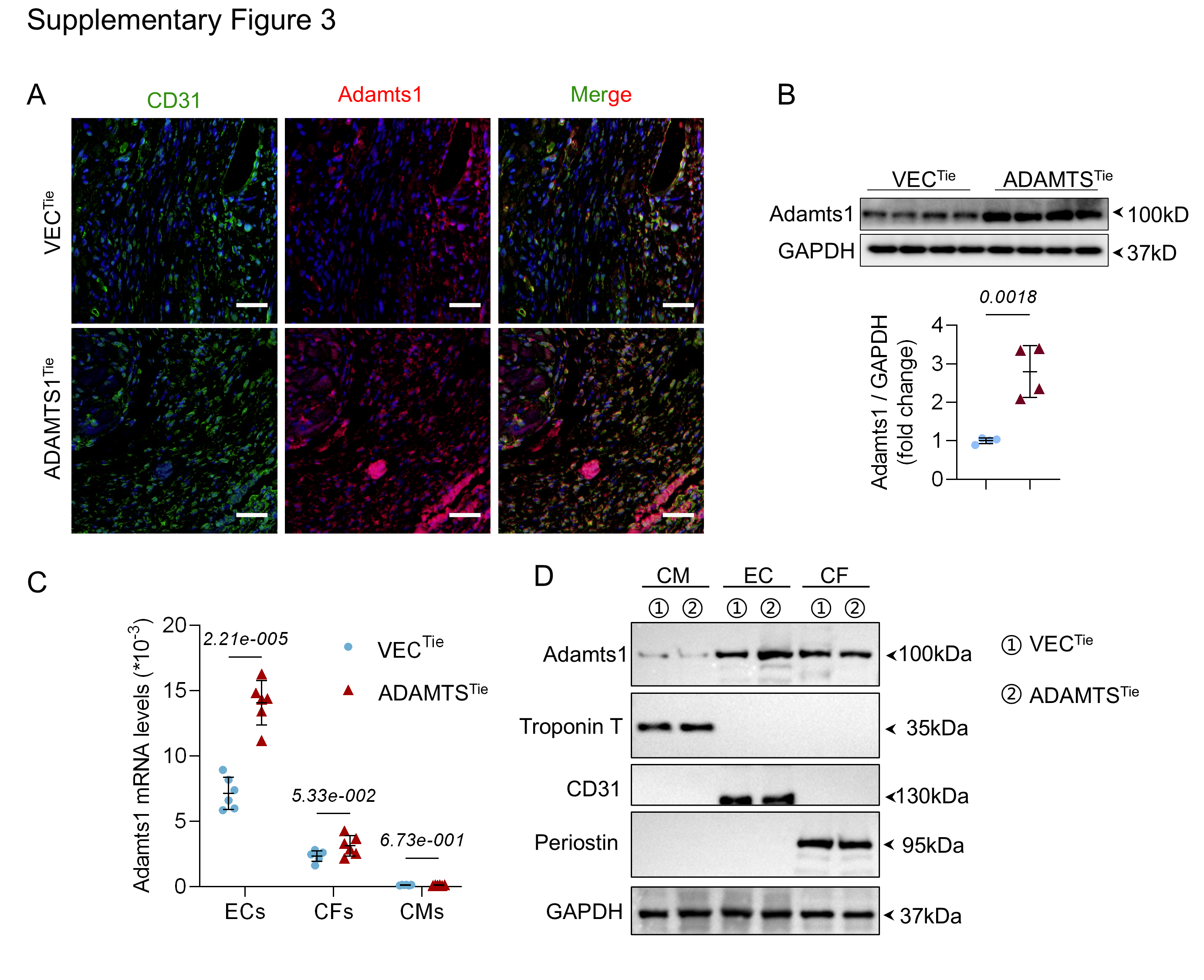


#### Supplementary Figure 3. Validation of Adamts1 Overexpression in Endothelial Cell-Specific Transgenic Mice

1. Immunofluorescence staining showing Adamts1 expression and co-localization with endothelial marker CD31 in VEC^Tie^ and ADAMTS1^Tie^ mice.

(B)Western blot analysis demonstrating Adamts1 overexpression efficiency in ADAMTS1^Tie^ mice (n=4 per group; t=5.291).

**(C)** *ADAMTS1* transcript levels in isolated ECs, CFs, and CMs in VEC^Tie^ and ADAMTS1^Tie^ mice (n=6 per group). t (ECs)=8.069, t (CFs)=2.196, t (CMs)=0.000.

**(D)** Western blot analysis showing cell-type-specific markers and Adamts1 expression in isolated ECs, CFs, and CMs from VEC^Tie^ and ADAMTS1^Tie^ mice. Statistical analyses were performed using multiple unpaired t-tests with two-stage step-up method of Benjamini, Krieger, and Yekutieli correction (B) and Student's t-test (C). Data are presented as mean±SD. Adamts1, a disintegrin and metalloproteinase with thrombospondin motif 1; CMs, cardiomyocytes; CFs, cardiac fibroblasts; ECs, endothelial cells.

####
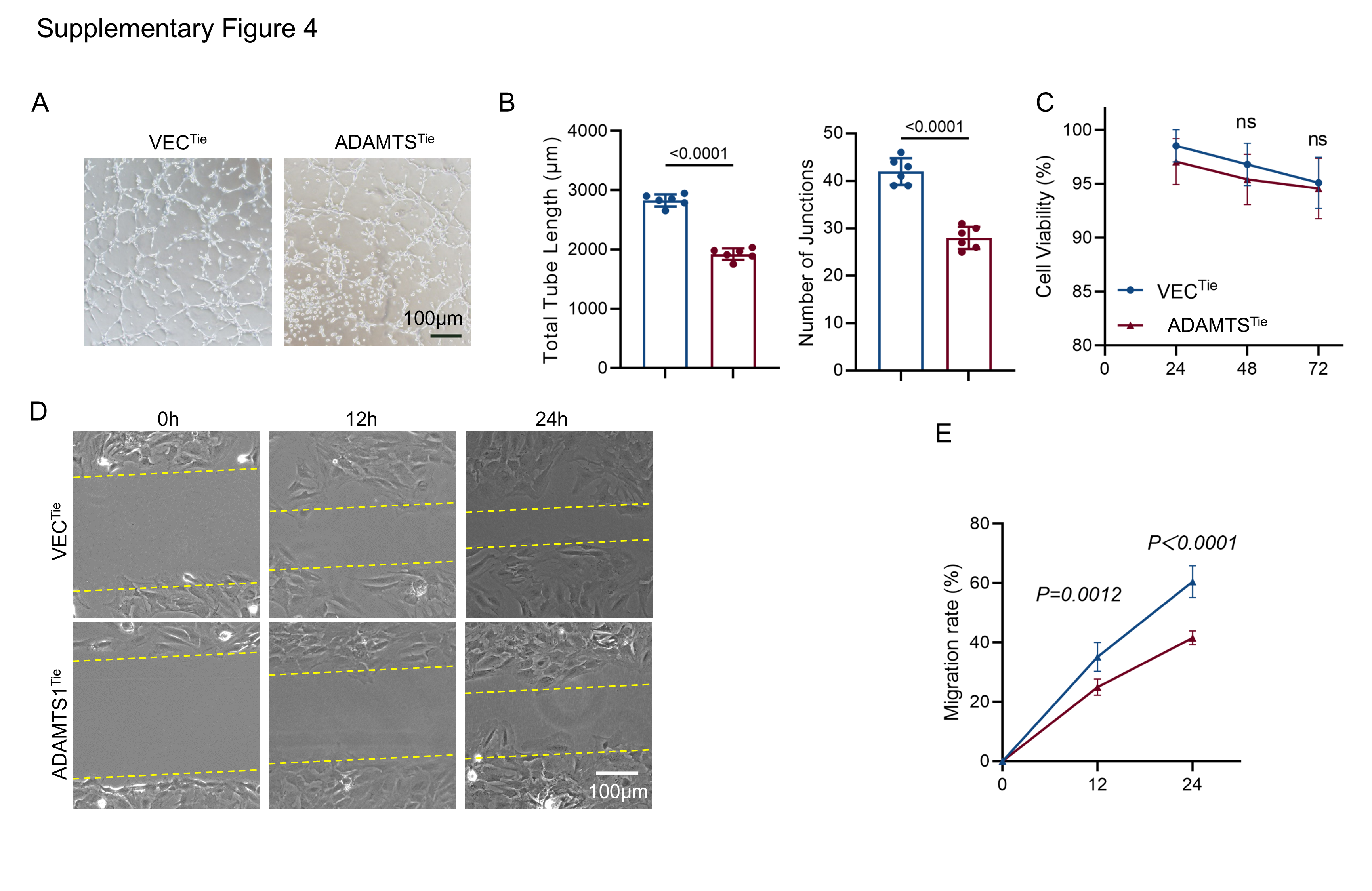
Supplementary Figure 4. Functional characterization of endothelial cells following ADAMTS1 overexpression.

1. Representative images of tube formation assay comparing VEC^Tie^ and ADAMTS1^Tie^ endothelial cells. Scale bar = 100 μm.
2. Quantitative analysis of tube formation parameters including total tube length (left) and number of junctions (right) in VEC^Tie^ and ADAMTS1^Tie^ endothelial cells (n=6 per group).
3. Cell viability assay showing percentage of viable cells over time (24, 48, and 72 hours) in VEC^Tie^ and ADAMTS1^Tie^ endothelial cells (n=6 per group). ns, not significant.
4. Representative images of wound healing migration assay at 0, 12, and 24 hours post-scratch. Yellow dashed lines indicate the wound boundaries. Scale bar = 100 μm.
5. Quantitative analysis of migration rate percentage over time (0, 12, and 24 hours) comparing VEC^Tie^ and ADAMTS1^Tie^ endothelial cells (n=6 per group).

Statistical analyses were performed using Student's t-test (**B, C, E**). Data are presented as mean±SD.

**
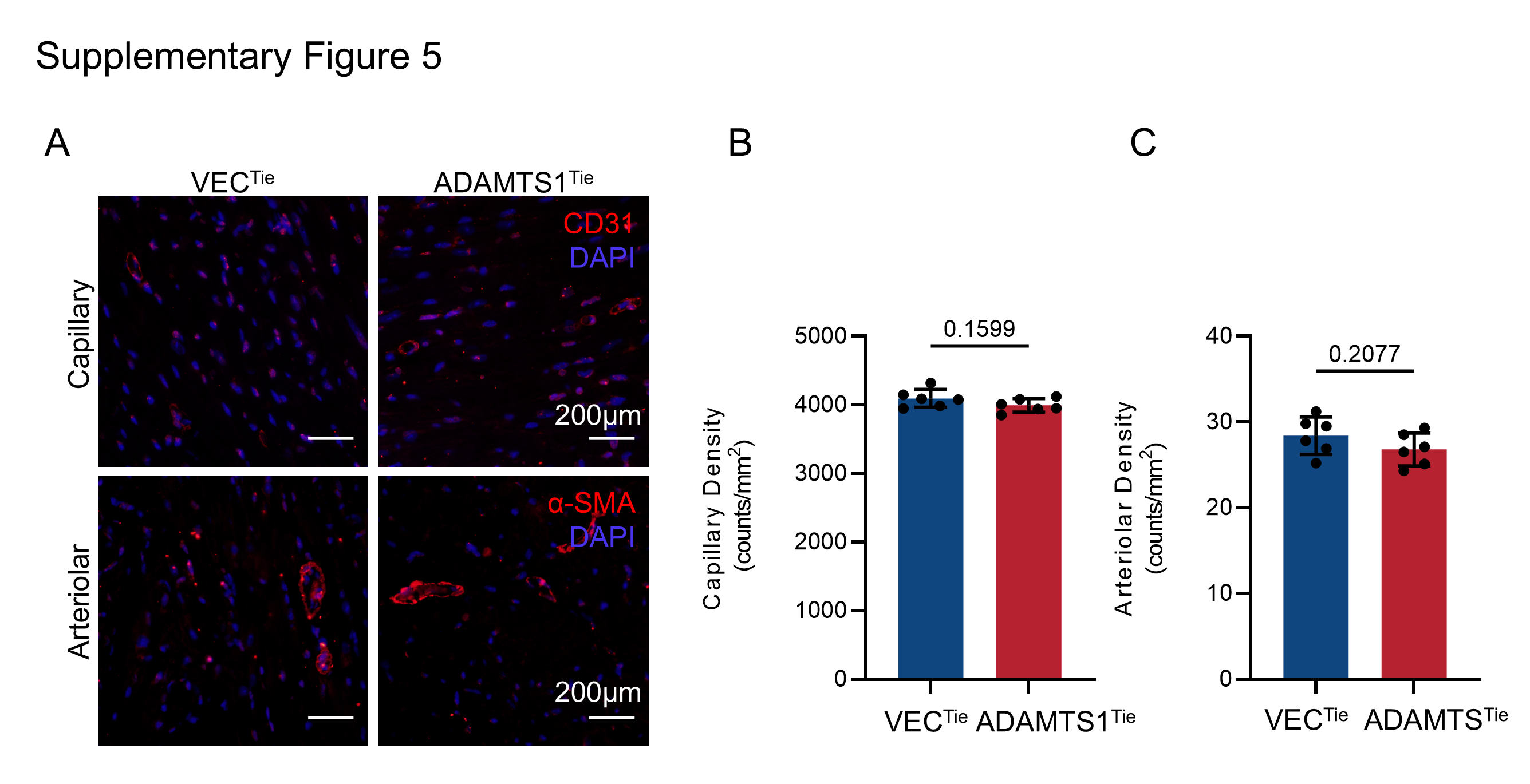
Supplementary Figure 5. Assessment of angiogenesis and vascular formation following Adamts1 overexpression.**

**(A)** Representative immunofluorescence images of cardiac tissue sections from VEC^Tie^ and ADAMTS1^Tie^ mice. Upper panels show capillary regions stained for CD31 (endothelial cell marker), and lower panels show arteriolar regions stained for α-SMA (smooth muscle cell marker). DAPI (blue) marks nuclei. Scale bars = 200 μm.

**(B)** Quantitative analysis of capillary density (capillaries per mm²) in cardiac tissue from VEC^Tie^ and ADAMTS1^Tie^ mice (n=6 per group).

**(C)** Quantitative analysis of arteriolar density (arterioles per mm²) in cardiac tissue from VEC^Tie^ and ADAMTS1^Tie^ mice (n=6 per group).

Statistical analyses were performed using unpaired t-test. Data are presented as mean±SD.


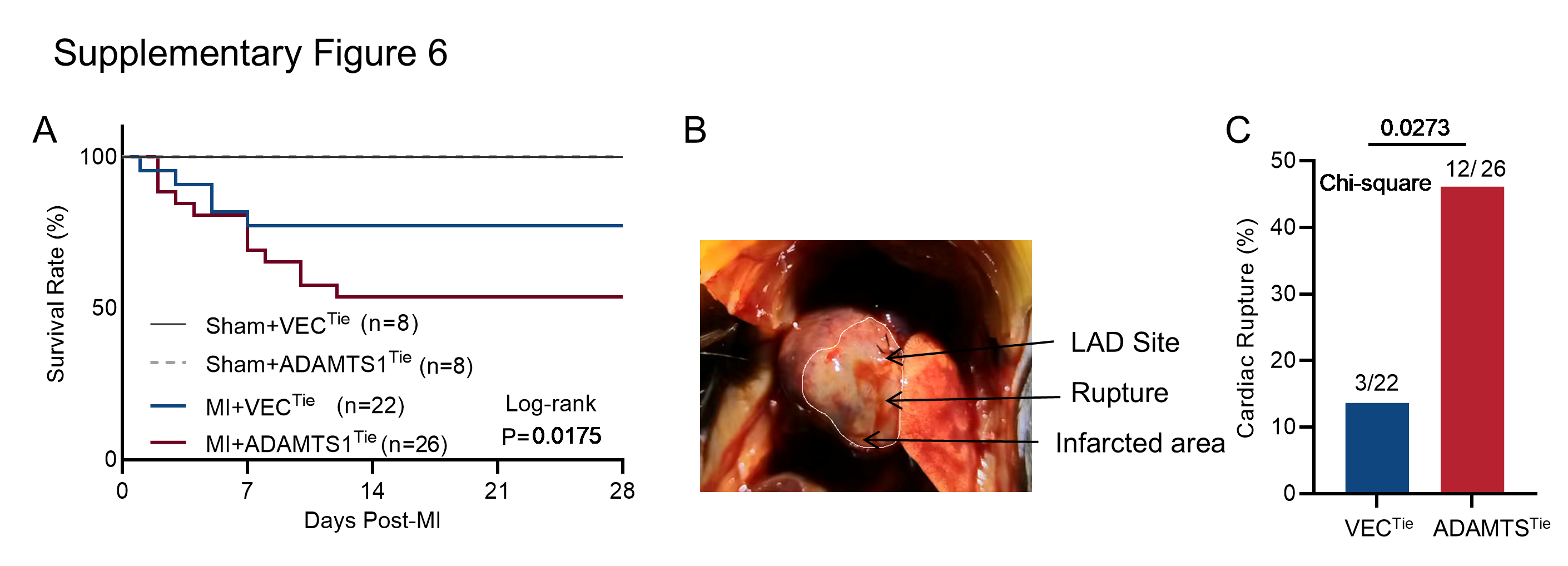


**Supplementary Figure 6. Survival analysis and cardiac rupture assessment following Adamts1 overexpression post-myocardial infarction.**

**(A)** Kaplan-Meier survival curves showing 28-day survival rates in Sham+VEC^Tie^ (n=8), Sham+ADAMTS1^Tie^ (n=8), MI+VEC^Tie^ (n=22), and MI+ADAMTS1^Tie^ (n=26). Statistical analysis performed using Log-rank test (P=0.0175).

**(B)** Representative macroscopic image of cardiac rupture in an ADAMTS1^Tie^ mouse post-MI, showing the left anterior descending (LAD) coronary artery ligation site, rupture location, and infarcted area.

**(C)** Quantitative analysis of cardiac rupture incidence comparing VEC^Tie^ (3/22, 13.6%) and Adamts1^Tie^ (12/26, 46.2%) groups. Numbers above bars indicate rupture cases/total cases.

Statistical analyses were performed using Log-rank test (**A**) and Chi-square test (**C**). Data are presented as survival percentages (A) and incidence rates (**C**).


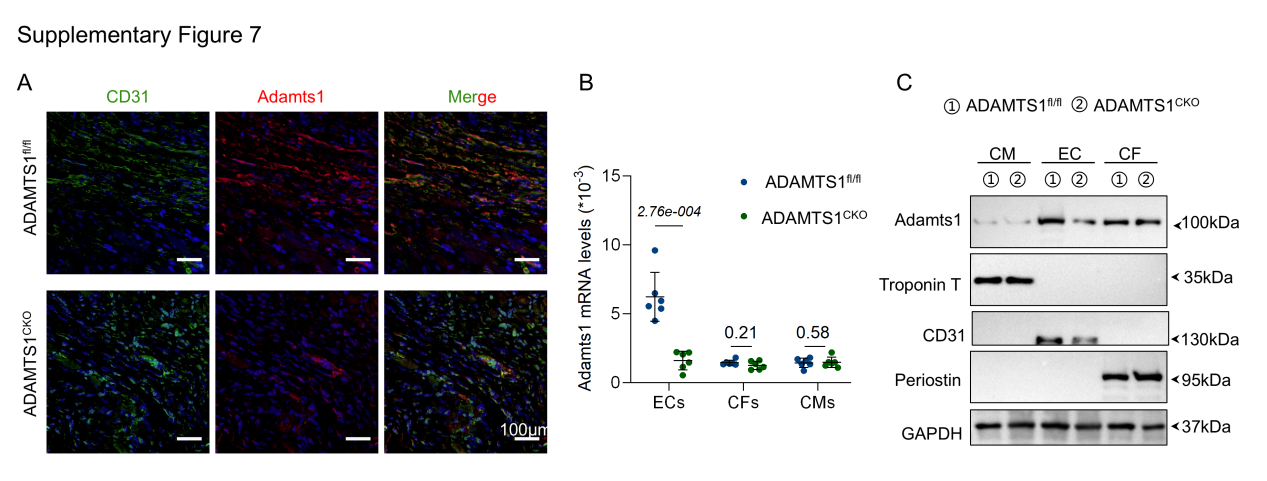


**Supplementary Figure 7. Validation of Endothelial Cell-specific Adamts1 Conditional Knockout Efficiency.**

**(A)** Representative immunofluorescence images showing co-localization of CD31 (endothelial cell marker, green) and Adamts1 (red) in cardiac tissue sections from ADAMTS1^fl/fl^ and ADAMTS1^CKO^ mice. DAPI (blue) indicates nuclei. Scale bars = 100 μm.

**(B)** qRT-PCR analysis of Adamts1 mRNA expression levels in isolated cardiac cell populations from ADAMTS1^fl/fl^ and ADAMTS1^CKO^ mice, including endothelial cells (ECs), cardiac fibroblasts (CFs), and cardiomyocytes (CMs).(n=6 per group).

**(C)** Western blot analysis showing cell-type-specific markers and Adamts1 protein expression in isolated cardiomyocytes (CM), endothelial cells (EC), and cardiac fibroblasts (CF) from ADAMTS1^fl/fl^ and ADAMTS1^CKO^ mice.

Statistical analyses were performed using unpaired t-test. Data are presented as mean±SD.


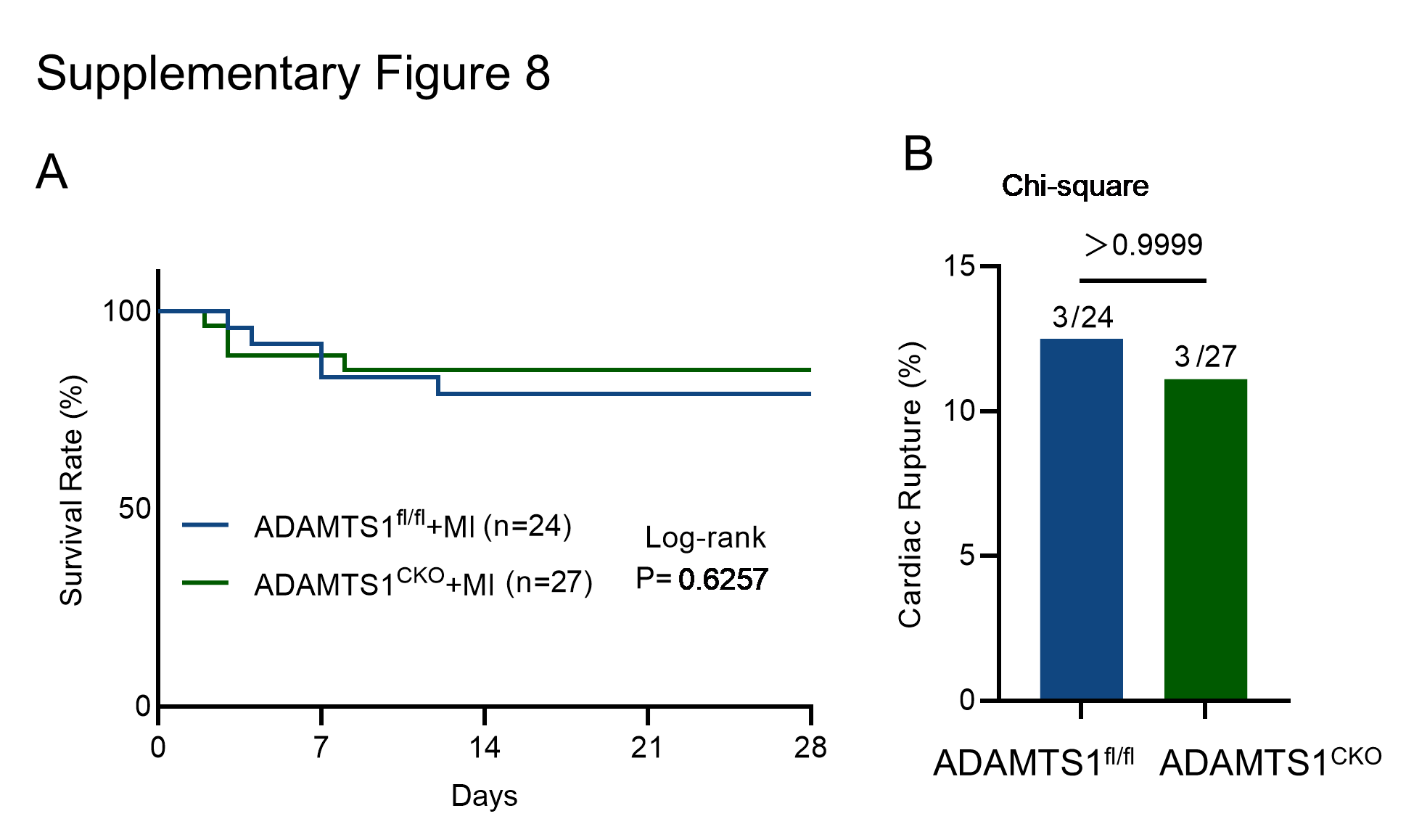


**Supplementary Figure 8. Survival Analysis and Cardiac Rupture Assessment in Adamts1 Conditional Knockout Mice Post-MI**

**(A)** Kaplan-Meier survival curves showing 28-day survival rates comparing ADAMTS1^fl/fl^+MI (n=24) and ADAMTS1^CKO^+MI (n=27) groups. Statistical analysis performed using Log-rank test (P=0.6257).

**(B)** Quantitative analysis of cardiac rupture incidence comparing ADAMTS1^fl/fl^+MI (3/24, 12.5%) and ADAMTS1^CKO^+MI (3/27, 11.1%) groups. Numbers above bars indicate rupture cases/total cases.

Statistical analyses were performed using Log-rank test (**A**) and Chi-square test (**B**).


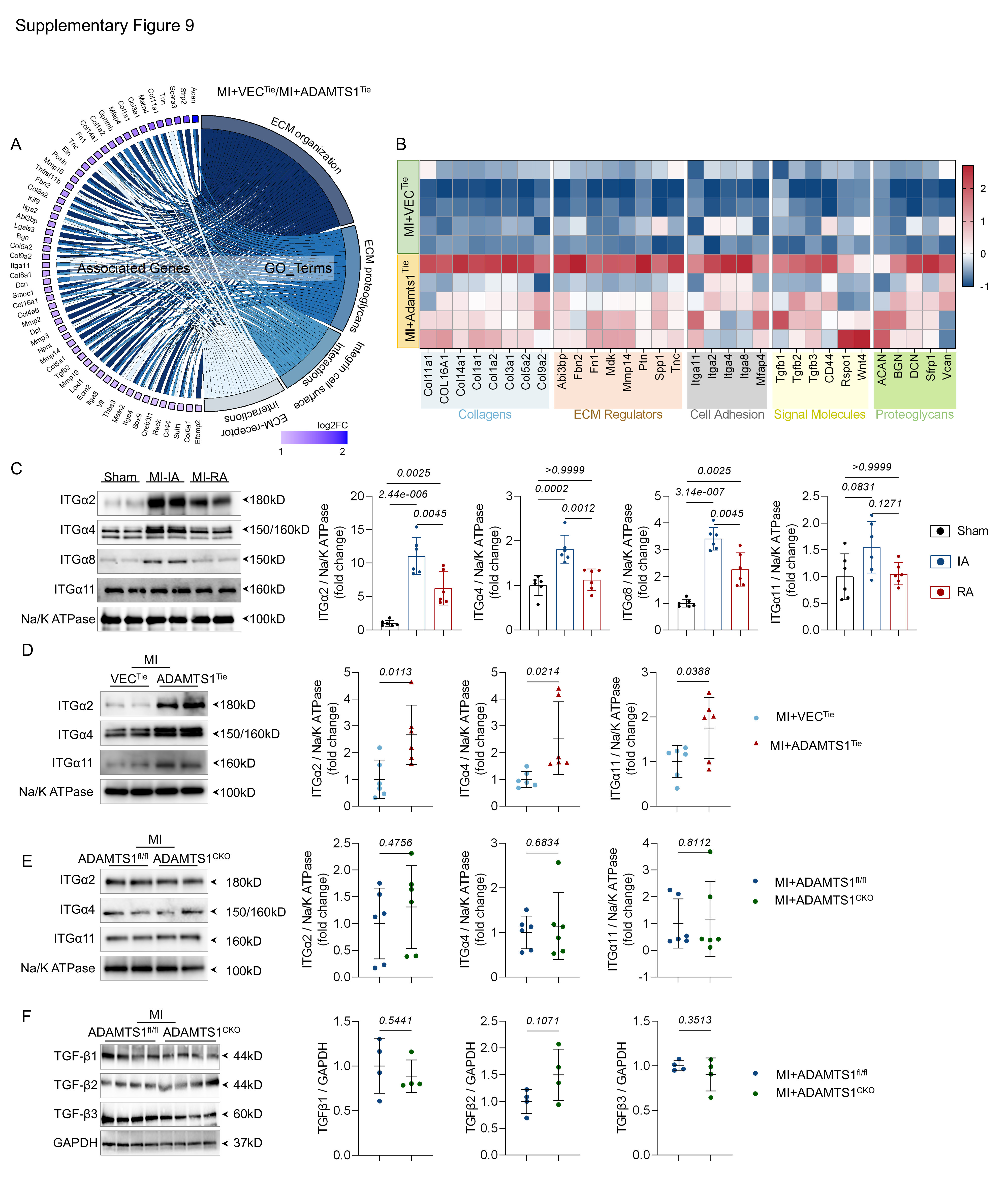


#### Supplementary Figure 9. Integrin α8 Signaling, but not Other Integrin α or TGF-β Signaling, Responds to Adamts1

**(A)** Chord diagram illustrating associations between genes and enrichment pathways (n=5).

**(B)** Heatmap depicting significantly altered genes involved in integrin cell surface interactions in ADAMTS1^Tie^ and VEC^Tie^ mice post-MI. Genes are categorized as collagen, ECM regulators, cell adhesion, signal molecules, and proteoglycans (n=3 per group).

**(C)** Western blot analysis of ITGα family expression in Sham and MI mice (n=6 per group). Statistical significance: ITGα2 (F(2,15)=32.32, P=3.65×10⁻⁶), ITGα4 (F(2,15)=16.66, P=0.0002), ITGα8 (F(2,15)=44.69, P=4.80×10⁻⁷), ITGα11 (F(2,15)=3.637, P=0.0515).

**(D)** Western blot analysis of ITGα2, α4, and α11 expression in Adamts1 overexpression models (n=6 per group). Adamts1 overexpression: ITGα2 (t=3.098), ITGα4 (t=2.725), ITGα11 (t=2.378);

**(E)** Western blot analysis of ITGα2, α4, and α11 expression in Adamts1 deficiency models (n=6 per group). Adamts1 deletion: ITGα2 (t=0.7412), ITGα4 (t=0.4200), ITGα11 (t=0.2453).

**(F)** Western blot analysis of TGF-β family expression in ADAMTS1^CKO^ and ADAMTS1^fl/fl^ mice post-MI (n=4 per group). TGF-β1 (t=0.6428), TGF-β2 (t=1.894), TGF-β3 (t=1.010).

Statistical analyses were performed using one-way ANOVA followed by Bonferroni post hoc test (**C**) and Student's t-test (**D-F**). Data are presented as mean±SD. Adamts1, a disintegrin and metalloproteinase with thrombospondin motif 1; VEC, vector; MI , myocardial infarction; Col, collagen; Abi3bp, ABI family member 3 binding protein; Fbn2, fibrillin 2; Fn1, fibronectin 1; Mdk, midkine; Mmp14, matrix metallopeptidase 14; Ptn, pleiotrophin; Spp1, secreted phosphoprotein 1; Tnc, tenascin C; ITGα, integrin subunit α; Mfap4, microfibril associated protein 4; Tgfb (), transforming growth factor beta; Rspo1, r-spondin 1; Wnt4, wnt family member 4; ACAN, aggrecan; BGN, biglycan; DCN, decorin; Sfrp1, secreted frizzled related protein 1; Vcan, versican; IA, infarct area; RA, remote infarct area; CKO, conditional knockout.


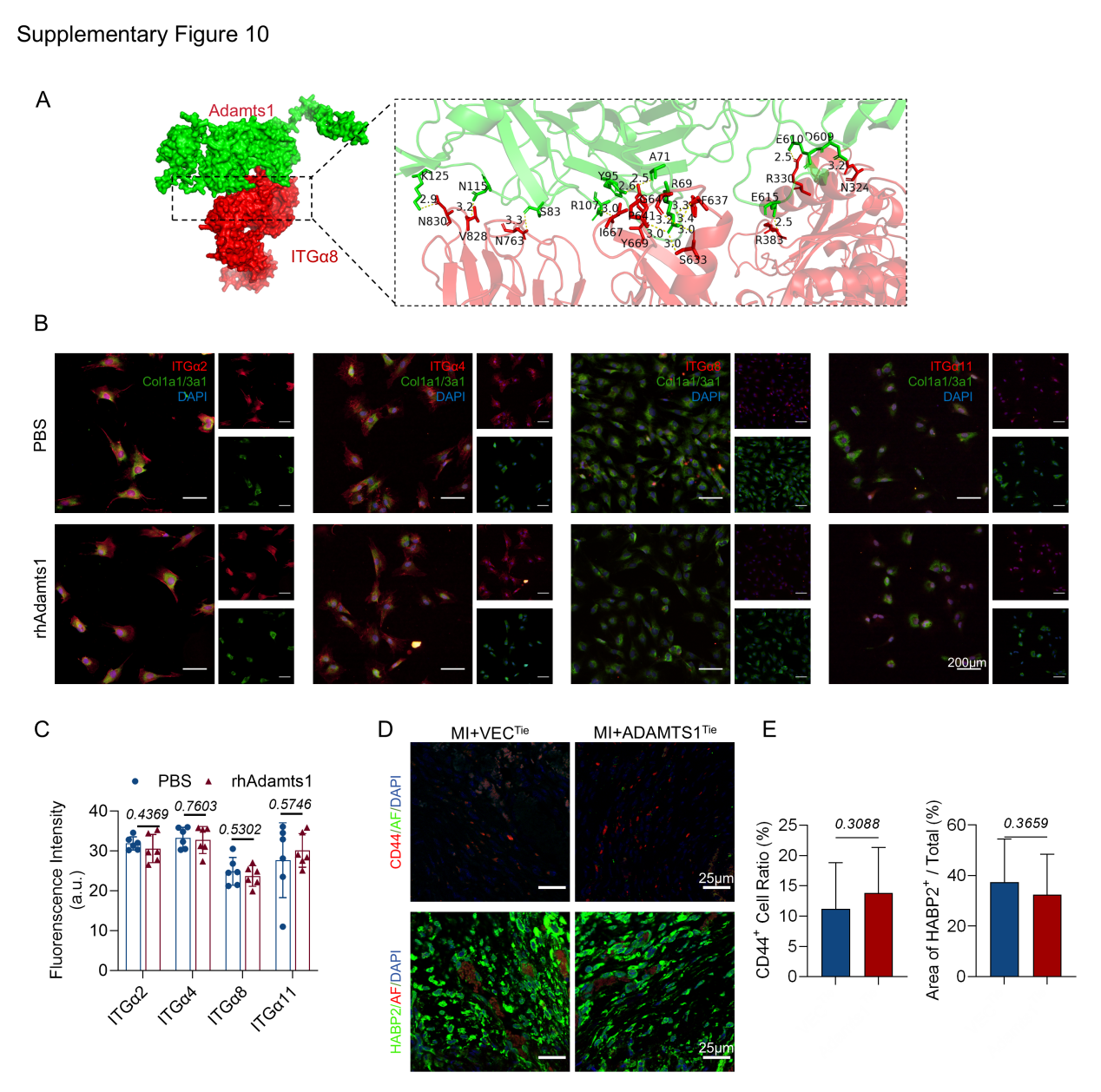


#### Supplementary Figure 10. Absence of Direct Activation of Integrin α by Adamts1

**(A)** Protein interaction prediction based on ZDOCK for human Adamts1 (PDB ID: Q9UHI8) and human ITGα8 (PDB ID: P53708). The docking score is -296.2 with a confidence interval of 0.949. Polar bonds formed between Adamts1 and ITGα8 at the protein-protein interaction interface are detailed in Supplementary Table 7.

**(B-C)** Immunofluorescence staining of cardiac fibroblasts treated with PBS or recombinant human Adamts1 protein, stained for ITGα2, ITGα4, ITGα8, and ITGα11, with quantitative analysis (n=5 per group). Statistical analysis: ITGα2 (t=0.8098), ITGα4 (t=0.3135), ITGα8 (t=0.6502), ITGα11 (t=0.5802).

**(D-E)** Immunofluorescence staining of cardiac tissue from VEC^Tie^ and ADAMTS1^Tie^ mice for CD44 and HABP2 (hyaluronic acid detection) post-MI, with quantitative analysis (n=6 per group). Statistical analysis: CD44 (t=1.256), HABP2 (t=0.9157). Data are presented as mean±SD. Statistical analyses were performed using unpaired t-test (C,E). Adamts1, a disintegrin and metalloproteinase with thrombospondin motif 1; HABP2, hyaluronic acid-binding protein 2; ITGα, integrin subunit α; MI, myocardial infarction; VCAN, versican.


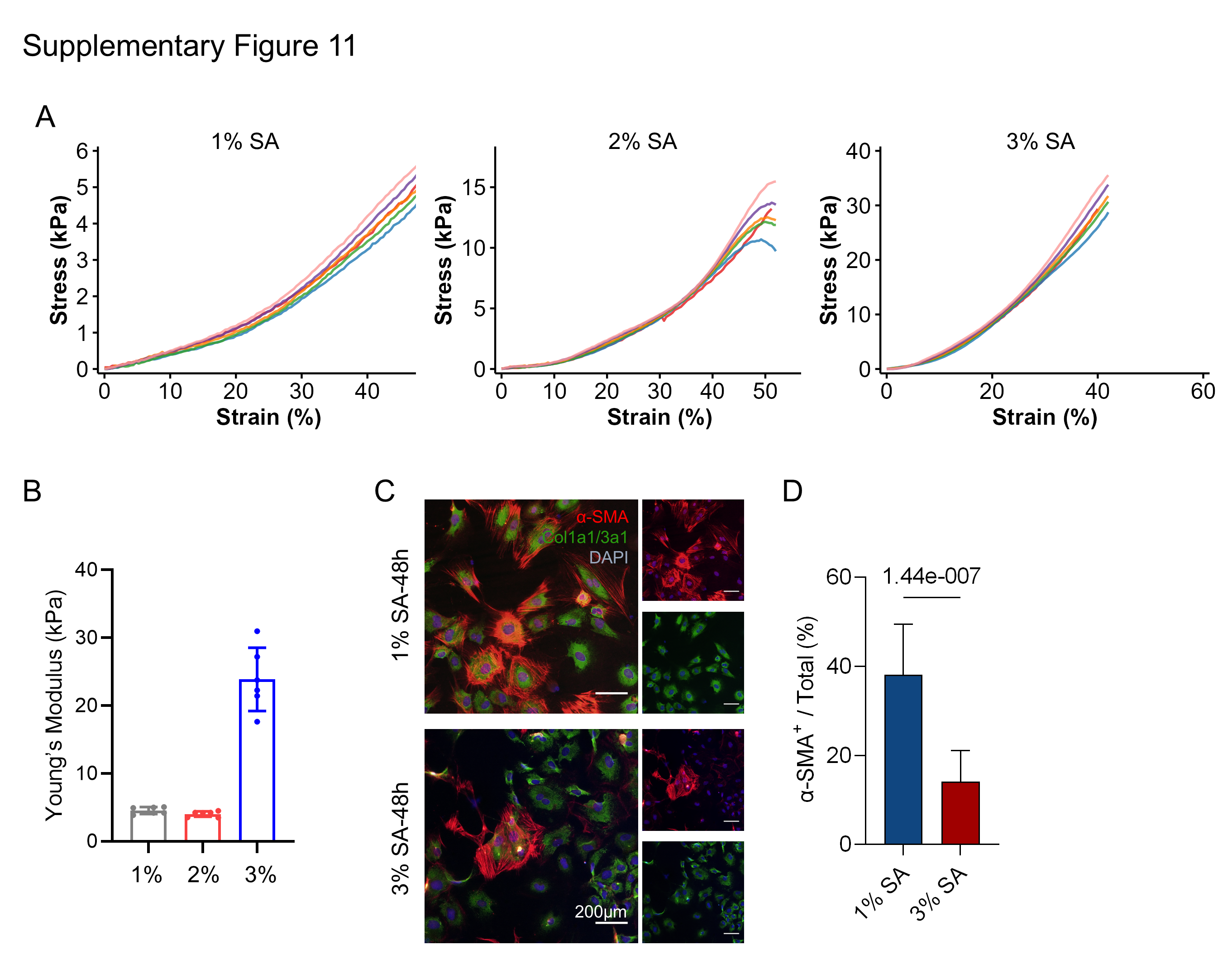


**Supplementary Figure 11. Mechanical Characterization of Sodium Alginate Hydrogels and Cardiac Fibroblast Response.**

**(A)** Stress-strain curves for individual samples of sodium alginate hydrogels at 1%, 2%, and 3% concentrations, demonstrating reproducibility across multiple independent samples.

**(B)** Quantitative analysis of Young's modulus for 1%, 2%, and 3% sodium alginate hydrogels (n=6 per group).

**(C)** Representative immunofluorescence images of cardiac fibroblasts cultured on 1% SA and 3% SA hydrogels. Cells were stained for α-SMA (red, myofibroblast marker), and DAPI (blue, nuclei). Scale bar = 200 μm.

**(D)** Quantitative analysis of α-SMA^+^ cell percentage in cardiac fibroblasts cultured on 1% versus 3% sodium alginate hydrogels (n=7 per group; t=6.962).

Statistical analysis was performed using Student's t-test. Data are presented as mean±SD.

**
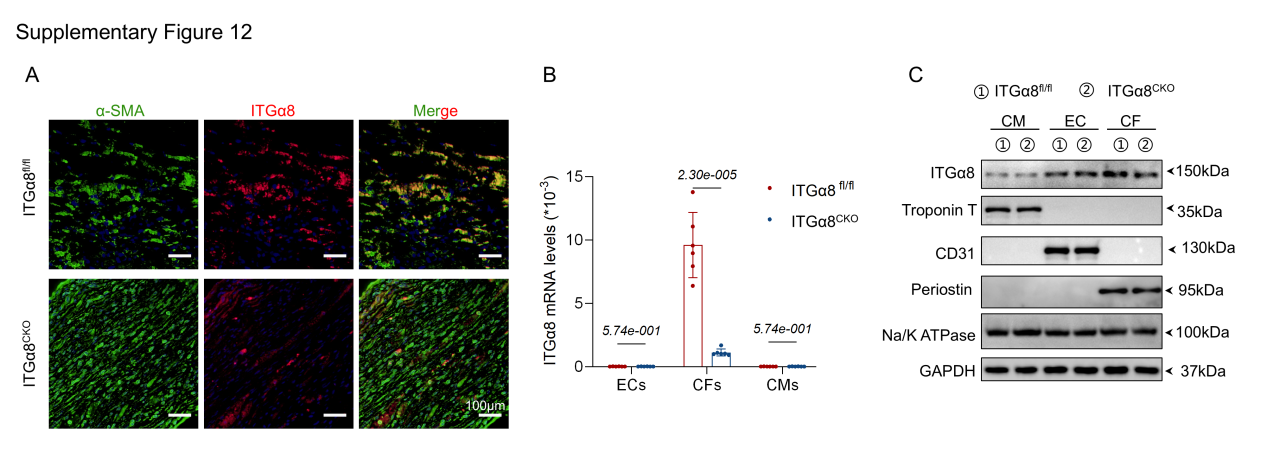
**

**Supplementary Figure 12. Validation of Cardiac Fibroblast-Specific ITGα8 Conditional Knockout Efficiency**

**(A)** Representative immunofluorescence images showing co-localization of α-SMA (green, myofibroblast marker) and ITGα8 (red) in cardiac tissue sections from ITGα8fl/fl and ITGα8^CKO^ mice. DAPI (blue) indicates nuclei. Scale bars = 100 μm.

**(B)** qRT-PCR analysis of ITGα8 mRNA expression levels in different cardiac cell populations isolated from Col1a2-CreER/ITGα8^fl/fl^ (ITGα8^CKO^) and ITGα8^fl/fl^ mice. Cell populations include CM, EC, and CF (n=6 per group).

**(C)** Western blot analysis showing cell-type-specific markers and ITGα8 protein expression in isolated CM, EC, and CF from /ITGα8^fl/fl^ and ITGα8^CKO^ mice. Troponin T (cardiomyocyte marker), CD31 (endothelial cell marker), and Periostin (fibroblast marker) were used for cell type identification. GAPDH serves as loading control.

Statistical analyses were performed using unpaired t-test. Data are presented as mean±SD. ITGα8, integrin subunit α8; CKO, conditional knockout; CFs, cardiac fibroblasts; CMs, cardiomyocytes; ECs, endothelial cells.


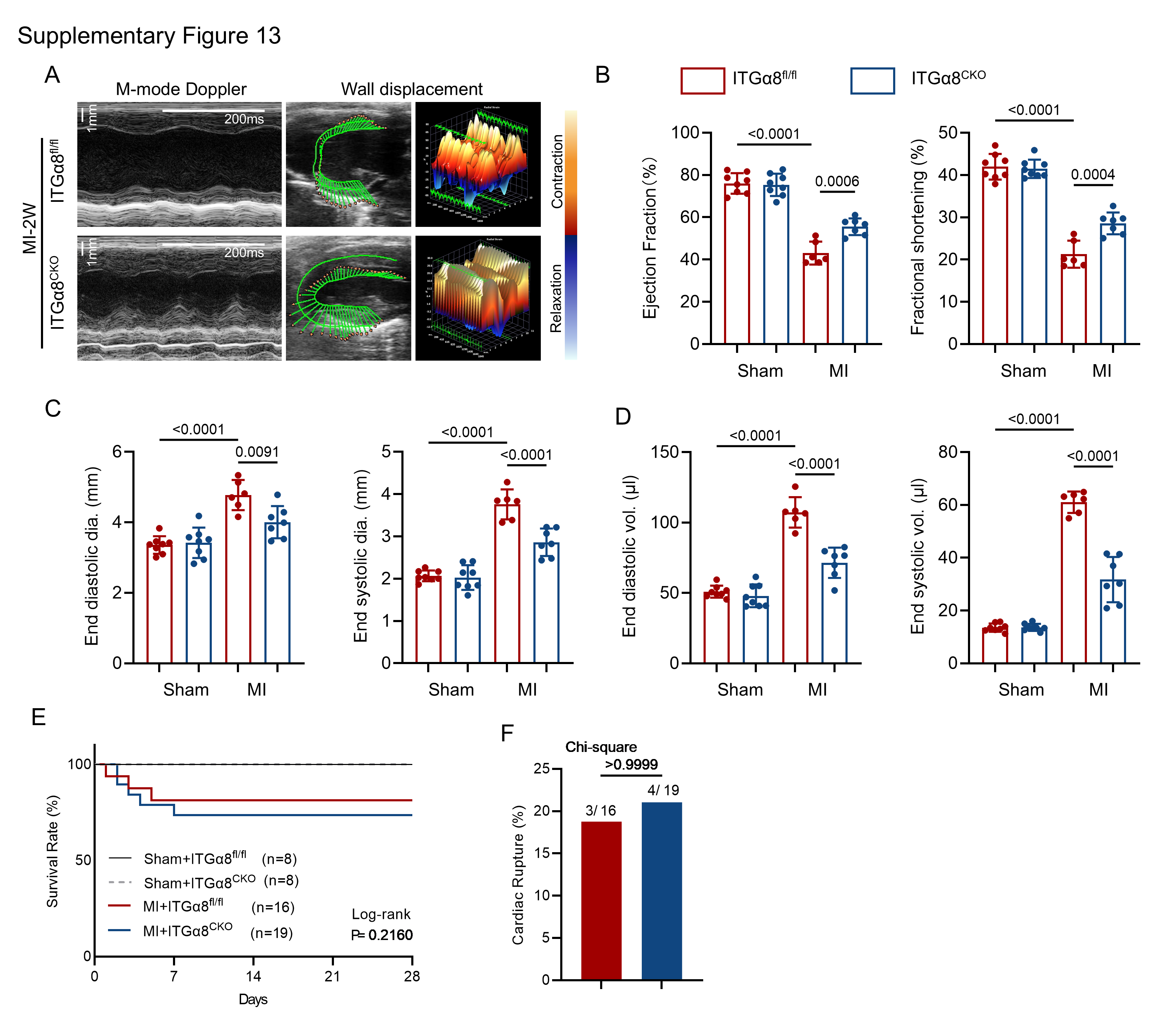


#### Supplementary Figure 13. Integrin α8 Deficiency Attenuates Cardiac Dysfunction Post-MI

#### (A) Representative M-mode, B-mode, and 3D speckle tracking ultrasound images showing cardiac function in ITGα8^fl/fl^ and ITGα8^CKO^ mice post-MI.

#### (B-D) Quantitative analysis of echocardiographic parameters at 2 weeks post-MI in ITGα8^CKO^ and ITGα8 mice: B, ejection fraction (EF) and fractional shortening (FS); C, end-diastolic diameter (EDD) and end-systolic diameter (ESD); D, end-diastolic volume (EDV) and end-systolic volume (ESV). Statistical significance: EF (t=4.809), FS (t=4.539), EDD (t=3.119), ESD (t=4.776), EDV (t=3.094), ESV (t=4.714).

(**E)** Kaplan-Meier survival curves showing 28-day survival rates in four experimental groups: Sham+ITGα8^fl/fl^ (n=8), Sham+ITGα8^CKO^ (n=8), MI+ITGα8^fl/fl^ (n=16), and MI+ITGα8^CKO^ (n=19). Statistical analysis performed using Log-rank test (P=0.2160).

**(F)** Quantitative analysis of cardiac rupture incidence comparing MI+ITGα8^fl/fl^ (3/16, 18.8%) and MI+ITGα8^CKO^ (4/19, 21.1%) groups. Numbers above bars indicate rupture cases/total cases. Statistical analysis performed using Chi-square test (P>0.9999).

####
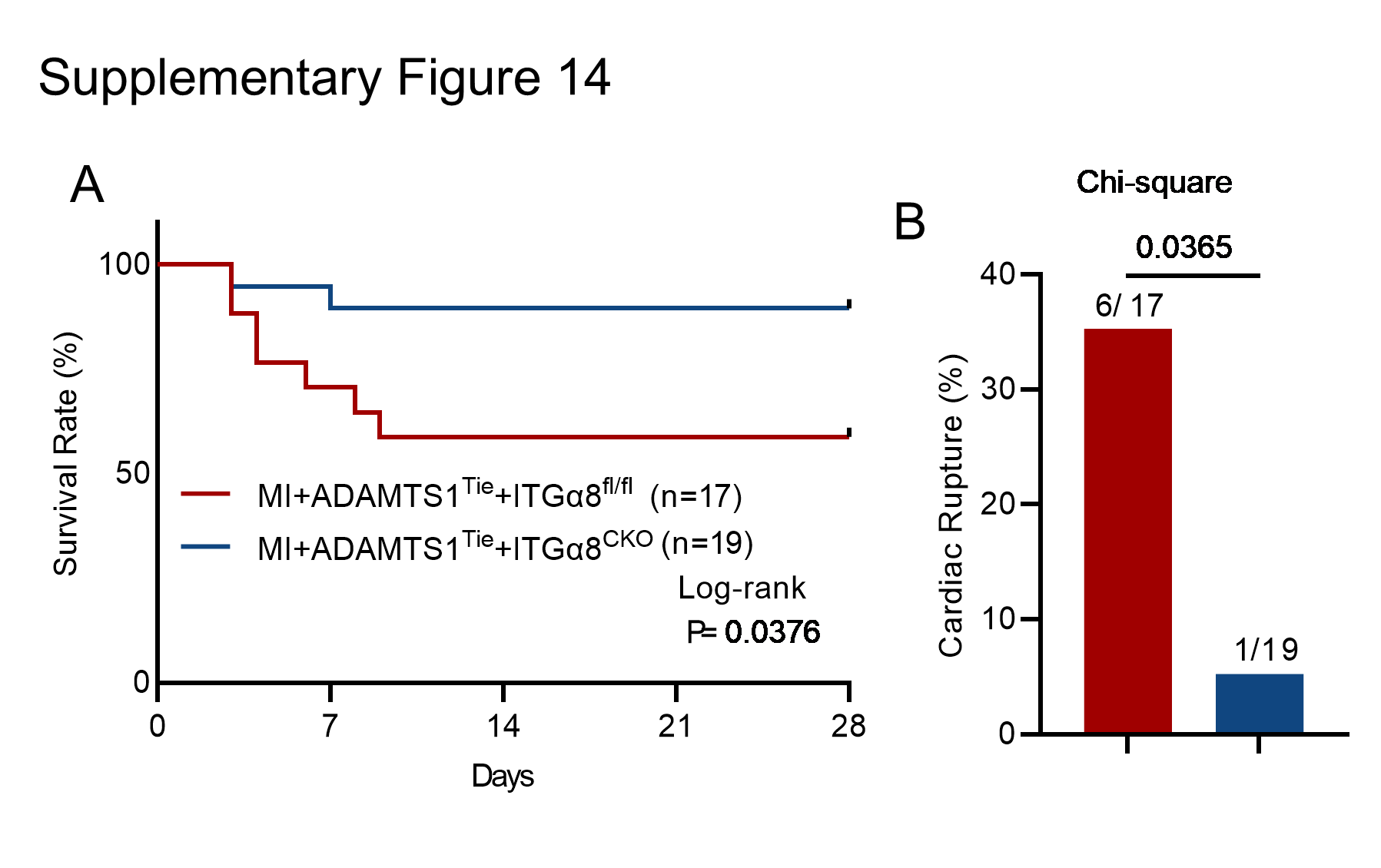


#### Supplementary Figure 14. ITGα8 deficiency improves survival and reduces cardiac rupture in Adamts1 overexpression mice post-myocardial infarction.

#### (A) Kaplan-Meier survival curves showing 28-day survival rates comparing MI+ADAMTS1^Tie^+ITGα8^fl/fl^ (n=17) and MI+ADAMTS1^Tie^+ITGα8^CKO^ (n=19) groups. Statistical analysis performed using Log-rank test

#### (B) Quantitative analysis of cardiac rupture incidence comparing MI+ADAMTS1^Tie^+ITGα8^fl/fl^ (6/17, 35.3%) and MI+ADAMTS1^Tie^+ITGα8^CKO^ (1/19, 5.3%) groups. Numbers above bars indicate rupture cases/total cases.

#### Data are presented as survival percentages (A) and incidence rates (B).

#### Supplementary Table 1

The detail information of human sample donors

| Group | No. | Age (years) | Gender | EF % | LVDD (mm) | NT-proBNP (pg/ml) | cTnI (ng/ml) |
| --- | --- | --- | --- | --- | --- | --- | --- |
| Health | 1 | 29 | Male | 66 | 44 | 15.00 | ＜0.006 |
| Health | 2 | 47 | Female | 60 | 48 | 38.00 | 0.034 |
| Ischemic | 1 | 72 | Male | 46 | 60 | 10164.00 | 25.118 |
| Ischemic | 2 | 67 | Female | 40 | 64 | 2195.00 | 1.550 |
| Ischemic | 3 | 65 | Female | 46 | 67 | 4498.00 | 0.141 |
| Ischemic | 4 | 79 | Male | 32 | 60 | 35614.00 | 2.676 |

EF, ejection fraction; LVDD, left ventricular end-diastolic diameter; NT-proBNP, N-terminal pro-brain natriuretic peptide; cTnI, Cardiac troponin I.

#### Supplementary Table 2

Gene_Count matrix of Figure 1C. All data are scaled by row.

| Sample_name/ | Sham_1 | Sham_2 | Sham_3 | Sham_4 | Sham_5 | MI_1 | MI_2 | MI_3 | MI_4 | MI_5 |
| --- | --- | --- | --- | --- | --- | --- | --- | --- | --- | --- |
| Group | Sham | | | | | MI | | | | |
| \gene_name |  | | | | | | | | | |
| Acan | 0 | 2 | 5 | 0 | 4 | 34 | 19 | 17 | 23 | 23 |
| Bgn | 5996 | 6935 | 5792 | 10543 | 7506 | 16924 | 9760 | 14151 | 14731 | 15678 |
| Col11a1 | 0 | 2 | 0 | 0 | 0 | 230 | 19 | 27 | 73 | 58 |
| Col12a1 | 26 | 56 | 26 | 18 | 78 | 742 | 195 | 433 | 242 | 476 |
| Col14a1 | 328 | 416 | 297 | 232 | 802 | 2006 | 905 | 1426 | 1986 | 1928 |
| Col1a1 | 3701 | 4167 | 3424 | 2793 | 4902 | 18347 | 9226 | 12962 | 16848 | 14904 |
| Col1a2 | 4232 | 4918 | 4326 | 2049 | 6408 | 23511 | 9982 | 14260 | 20392 | 18626 |
| Col3a1 | 5551 | 7061 | 5764 | 3435 | 14376 | 46069 | 17479 | 25062 | 51322 | 34595 |
| Col5a2 | 732 | 1023 | 776 | 570 | 1953 | 5455 | 2016 | 2843 | 4869 | 3973 |
| Col5a3 | 898 | 764 | 902 | 843 | 2385 | 2707 | 2364 | 2213 | 3543 | 2130 |
| Col8a2 | 17 | 27 | 18 | 2 | 21 | 787 | 349 | 590 | 228 | 766 |
| Col9a2 | 17 | 6 | 7 | 5 | 3 | 52 | 30 | 39 | 25 | 44 |
| Comp | 119 | 218 | 98 | 41 | 120 | 1822 | 1331 | 3017 | 441 | 4402 |
| Eln | 1530 | 1401 | 1501 | 1280 | 2983 | 15209 | 7201 | 8848 | 11396 | 15962 |
| Emilin1 | 1224 | 1221 | 1258 | 311 | 1177 | 1590 | 2113 | 2452 | 1916 | 2467 |
| Fbn1 | 2021 | 2823 | 2168 | 2311 | 4791 | 7806 | 4510 | 6072 | 7586 | 6753 |
| Fbn2 | 43 | 38 | 24 | 39 | 112 | 225 | 100 | 121 | 311 | 168 |
| Fn1 | 1845 | 1977 | 1817 | 1794 | 3032 | 10463 | 4459 | 6235 | 7666 | 8192 |
| Impg2 | 0 | 2 | 0 | 3 | 17 | 8 | 2 | 6 | 12 | 0 |
| Mfap4 | 441 | 619 | 484 | 831 | 560 | 3685 | 1916 | 3390 | 2777 | 4055 |
| Mfap5 | 646 | 700 | 566 | 225 | 883 | 2899 | 1340 | 2170 | 2665 | 2232 |
| Nepn | 29 | 25 | 36 | 41 | 91 | 32 | 68 | 48 | 93 | 59 |
| Postn | 649 | 1004 | 796 | 766 | 2064 | 15003 | 2896 | 5900 | 10721 | 6740 |
| Spp1 | 12 | 14 | 8 | 8 | 11 | 168 | 40 | 52 | 235 | 81 |
| Tnc | 42 | 70 | 60 | 57 | 120 | 976 | 145 | 289 | 577 | 263 |
| Vcan | 167 | 370 | 249 | 144 | 685 | 1372 | 257 | 515 | 1540 | 501 |

Acan: Aggrecan, Bgn: Biglycan, Col: Collagen, Comp: Cartilage Oligomeric Matrix Protein, Eln: Elastin, Emilin1: Elastin Microfibril Interfacer 1, Fbn: Fibrillin, Fn1: Fibronectin 1, Impg2: Interphotoreceptor Matrix Proteoglycan 2, Mfap: Microfibril Associated Protein, Nepn: Nephrocan, Postn: Periostin, Spp1: Secreted Phosphoprotein 1, Tnc: Tenascin C, Vcan: Versican, MI: Myocardial Infarction

#### Supplementary Table 3

Proteomic Data matrix of Figure 6A. Differential expressed proteins of infarct scar in ADAMTS1^fl/fl^ and ADAMTS1^CKO^ mice. (Provided as a separate Excel file.)

#### Supplementary Table 4

DEGs of infarct scar in MI+VEC^Tie^ and MI+ADAMTS1^Tie^ mice related in Figure 6A. (Provided as a separate Excel file.)

#### Supplementary Table 5

Gene Primers

| Targets | Forward (F) /Reverse (R) Sequence |
| --- | --- |
| Adamts1 | F: 5'-GGAACCGTATGTGACCCCAG-3'  R: 5'-TGAGAATCGCCACTCACACC-3' |
| Adamts1-Homo sapiens | F: 5'-CCTGAGCAGAGTGAGCAACA-3'  R: 5'-CAGCGCAAAGTTGGAGACAC-3' |
| Adamts1-Rattus norvegicus | F: 5'-CAAACGAGTCCGCTACAGGT-3'  R: 5'-CCATCTACCACCTTGGGCTG-3' |
| Col1a1 | F: 5'-GCTTCACCTACAGCACCCTT-3'  R: 5'-GGTGGGAGGGAACCAGATTG-3' |
| Col1a2 | F: 5'-CACCCCAGCGAAGAACTCAT-3'  R: 5'-TCTCCTCATCCAGGTACGCA-3' |
| Col3a1 | F: 5'-ACGTAAGCACTGGTGGACAG-3'  R: 5'-GGAGGGCCATAGCTGAACTG-3' |
| Col5a2 | F: 5'-GAGCGTGGTCCTGTAGGTTC-3'  R: 5'-GGATTTCCTGTCAGACCCCG-3' |
| Col8a2 | F: 5'-AGAGCGACGCGGAGTT-3'  R: 5'-GCTGCACATACTTTACCGGG-3' |
| Col11a1 | F: 5'-TGGTGATCAGAATCAGAAGTTCG-3'  R: 5'-AGGAGAGTTGAGAATTGGGAATC-3' |
| Col12a1 | F: 5'-CCGTACAATGGGCAAGGCTA-3'  R: 5'-CTGCCGCGAGATTTCCATAC-3' |
| Col14a1 | F: 5'-TGAAGCACCCACAGCCATAG-3'  R: 5'-TTGCACTCCAGGCACCATAA-3' |
| Acan | F: 5'-ACCGTTGCAGACATTGACGA-3'  R: 5'-TAGCTCGGAAGGCATAAGCA-3' |
| Bgn | F: 5'-TGGGCACAAAGAGGGAGTAG-3'  R: 5'-GTGTGACAGAGTCCAGGTCG-3' |
| Comp | F: 5'-GCTCAAGGCTGTCAAGTCCT-3'  R: 5'-GGACCCTCATAGAACCGCAC-3' |
| Eln | F: 5'-CCTAGGGTGCAAAGGGTTGT-3'  R: 5'-TAAACCCAAAGAGCACACCAAC-3' |
| Emilin1 | F: 5'-TGCTGTCTGCTGACCATAGC-3'  R: 5'-CTCACTGTCCGAGTCACCAC-3' |
| Fbn1 | F: 5'-CTCAGCCACCAGAGGATCAC-3'  R: 5'-CCCCTCCAGCCTTAATCCAC-3' |
| Fbn2 | F: 5'-GGGGATGCAACGTGGAC-3'  R: 5'-AGCACTGTCGGAGACCAAAT-3' |
| Fn1 | F: 5'-CAACCCTGGGTATGACACCG-3'  R: 5'-GGCCCGGAACATGAGGATAG-3' |
| Impg2 | F: 5'-GCAGGGAACTCCAGTCACAA-3'  R: 5'-CTGAAGCACTGCTGGGGTAA-3' |
| Mfap4 | F: 5'-CGGCGTGTATCTCATCTACCC-3'  R: 5'-CCCCATGGCCCCACCTTAC-3' |
| Mfap5 | F: 5'-ACAGACGATACAGCTGGTGAC-3'  R: 5'-TACATGCGCCGTAAACTGGT-3' |
| Nepn | F: 5'-TGGGGCTCTCTCTTACCAATG-3'  R: 5'-AGCCAACAGTCCAGTGAAGT-3' |
| Postn | F: 5'-GGAAGGACCTGCAATGACGA-3'  R: 5'-CCACCTCCTGTGGAAATCCT-3' |
| Spp1 | F: 5'-TGGCTGAATTCTGAGGGACTAA-3'  R: 5'-CTGCTTCTGAGATGGGTCAGG-3' |
| Tnc | F: 5'-CAACTACAGCCTCCCCACAG-3'  R: 5'-ATGGCCGTGGATGCCTTC-3' |
| VCAN | F: 5'-AATGACGGTGGGTGTCACAA-3'  R: 5'-GCCCCGGAAGATCACATAGG-3' |
| GAPDH | F: 5'- AGGTCGGTGTGAACGGATTTG-3'  R: 5'-GGGGTCGTTGATGGCAACA-3' |
| GAPDH-[Rattus norvegicus](https://www.ncbi.nlm.nih.gov/datasets/taxonomy/10116) | F: 5'- CCGCATCTTCTTGTGCAGTG-3'  R: 5'- ACCAGCTTCCCATTCTCAGC-3' |
| GAPDH-Homo sapiens | F: 5'- AATGGGCAGCCGTTAGGAAA-3'  R: 5'- GCGCCCAATACGACCAAATC-3' |

Gene primers were obtained from the NCBI database via the Basic Local Alignment Search Tool (BLAST) and were used to detect the expression of the corresponding genes at the mRNA level. Except for specific labeling, the above primers are Mus Musculus derived. Acan: Aggrecan, Bgn: Biglycan, Col: Collagen, Comp: Cartilage Oligomeric Matrix Protein, Eln: Elastin, Emilin1: Elastin Microfibril Interfacer 1, Fbn: Fibrillin, Fn1: Fibronectin 1, Impg2: Interphotoreceptor Matrix Proteoglycan 2, Mfap: Microfibril Associated Protein, Nepn: Nephrocan, Postn: Periostin, Spp1: Secreted Phosphoprotein 1, Tnc: Tenascin C, Vcan: Versican, MI: Myocardial Infarction.

#### Supplementary Table 6

Primary Antibodies

| Targets | Vendor | Source | Catalog # | Dilution Proportion |
| --- | --- | --- | --- | --- |
| Adamts1 | Invitrogen  Abcam | Rabbit | 720329  ab39194 | WB: 1:1000  ISI: 1:100 |
| GAPDH | Cell Signaling Technology | Rabbit | 2118 | WB: 1:1000 |
| ITGα2 | ABclonal | Rabbit | A7629 | WB: 1:1000  ISI: 1:100 |
| ITGα4 | ABclonal | Rabbit | A0696 | WB: 1:1000  ISI: 1:100 |
| ITGα8 | Abcam | Mouse | ab243027 | WB: 1:1000  ISI: 1:200 |
| ITGα11 | ABclonal | Rabbit | A10084 | WB: 1:1000  ISI: 1:100 |
| Na/K ATPase | Cell Signaling Technology | Rabbit | 3010 | WB: 1:1000 |
| CD31 | Cell Signaling Technology | Mouse | 3528S | ISI: 1:100 |
| Col 1a1 | Invitrogen  Santa Cruz Biotechnology | Rabbit  Mouse | PA5-29569  sc-293182 | ISI: 1:100  ISI: 1:20 |
| Col 3a1 | Santa Cruz Biotechnology  Cell Signaling Technology | Mouse  Rabbit | sc-514601  30565 | ISI: 1:20  ISI: 1:100 |
| Vinmentin | Santa Cruz Biotechnology | Mouse | sc-6260 | ISI: 1:20 |
| Postn | Proteintech | Rabbit | 19899-1-AP | ISI: 1:100 |
| α-SMA | Abcam | Rabbit  Mouse | ab5694  ab7817 | ISI: 1:200 |
| BrdU | ABclonal | Mouse | A1482 | ISI: 1:100 |
| Decorin | ABclonal | Rabbit | A1669 | ISI: 1:100 |
| VCAN | ABclonal | Rabbit | A20278 | ISI: 1:100 |
| Versikine | ThermoFisher Scientific | Rabbit | PA1-1748A | ISI: 1:200 |
| TGF-β1 | Proteintech | Rabbit | 21898-1-AP | WB: 1:1000 |
| TGF-β2 | ThermoFisher Scientific | Rabbit | PA5-92040 | WB: 1:2000 |
| TGF-β3 | ThermoFisher Scientific | Rabbit | PA5-97064 | WB: 1:2000 |

#### Supplementary Table 7

Amino acid sites and bond lengths between Adamts1 and ITGα8 protein predicted with ZDOCK.

| Adamts1-residues | ITGα8-residues | Polar-Bond (Å) |
| --- | --- | --- |
| K125 | N830 | 2.9 |
| N125 | V828 | 3.2 |
| S83 | N763 | 3.3 |
| R107 | I667 | 3.0 |
| Y95 | Y669 | 2.6 |
| A71 | Y669 | 2.5 |
| R69 | P641 | 3.0 |
| R69 | G640 | 3.2 |
| R69 | S633 | 3.0 / 3.0 |
| R69 | F637 | 3.3 / 3.4 |
| E615 | R383 | 2.5 |
| E610 | R330 | 2.5 |
| D609 | N324 | 3.2 |

A: Alanine; D: Aspartic acid; E: Glutamic acid; F: Phenylalanine; G: Glycine; I: Isoleucine; K: Lysine; N: Asparagine; P: Proline; R: Arginine; S: Serine; V: Valine; Y: Tyrosine.
